# Estimating Mean Growth Trajectories When Measurements Are Sparse and Age Is Uncertain

**DOI:** 10.1002/ajpa.70358

**Published:** 2026-09-22

**Authors:** John A. Bunce, Caissa Revilla‐Minaya, Catalina I. Fernández

**Affiliations:** ^1^ Division of Anthropology American Museum of Natural History New York New York USA; ^2^ Department of Human Behavior, Ecology, and Culture Max Planck Institute for Evolutionary Anthropology Leipzig Germany; ^3^ Department of Anthropology Florida Atlantic University Boca Raton Florida USA

**Keywords:** age uncertainty, bioarcheology, children, growth, ontogeny

## Abstract

**Objectives:**

Comparing children's growth across the world and at different moments in history can yield insight into both health challenges and healthy morphological variation in humans. A difficulty of such comparative analyses is that, in marginalized populations, there are often logistical complications to obtaining repeat measures of individual children's height and weight. The problem is even more acute for historical populations: bioarcheological datasets comprise single measures of individuals at death. Additionally, for both contemporary and historical populations, there is often non‐trivial uncertainty about children's ages. Both of these factors complicate estimation of growth trajectories. Here we evaluate the degree to which we can accurately estimate a population‐mean growth trajectory using only a small number of (randomly) uncertain measurements, like those that compose many contemporary and bioarcheological datasets.

**Materials and Methods:**

We recently derived a causal model of human growth from fundamental principles of metabolism and allometry, permitting exploration of genetic and environmental contributions to children's growth. Here, we fit this model in a Bayesian framework to simulated cross‐sectional and longitudinal datasets of varying size, where age is uncertain.

**Results:**

For large‐scale comparisons, reasonably accurate population‐mean growth trajectories may be obtained from single height measures of 100 children of a given sex and a range of ages. However, detailed analyses of pubertal growth spurts and the metabolic and allometric parameters underlying growth require longitudinal datasets.

**Discussion:**

We conclude that this new model and estimation strategy constitute a potentially useful toolkit for comparing mean growth trajectories across contemporary and historical populations.

## Research Highlights

- Mean growth trajectories can be reasonably estimated from single height measures of 100 children of uncertain age using a Bayesian growth model.
- This allows exploration of growth in marginalized and archaeological populations with sparse data.

## 1 Introduction

The size and proportions of the human body exhibit considerable diversity, both geographically, and through history. Some of this diversity in body form may result from drift and natural selection of genetic variants (Lello et al. 2018; Zoccolillo et al. 2020) over evolutionary time in different populations distributed across a broad range of ecologies (Katzmarzyk and Leonard 1998). However, environmental factors such as food availability, pathogen exposure, and chronic stress, acting within an individual's lifetime, can also have profound effects on the shape of the body (Bogin 2022; Li et al. 2020; Stewart et al. 2013; Tanner et al. 1982). For this reason, height and weight at a given age are commonly used to diagnose health challenges, such as malnutrition (de Onis and Branca 2016; World Health Organization and United Nations Children's Fund 2009), that result from adverse environmental conditions. However, determining the relative contributions of genes and the environment to body form is difficult, which complicates formulating diagnoses and making healthcare decisions on the basis of comparisons of body size between populations with different genetic histories in different ecological contexts around the world (Hackman and Hruschka 2020; Hruschka 2021).

As an initial step toward addressing this problem, we have recently derived a new theoretical mathematical model of human height and weight growth from foundational principles of metabolism and allometry (Bunce et al. 2025). This new model complements other popular parametric (e.g., Jolicoeur et al. (1992)) and shape‐invariant (e.g., Cole et al. (2010), Nierop et al. (2016)) human growth models by, uniquely, permitting investigation of the metabolic and allometric causes of particular patterns of growth. Under an assumption that metabolic rates are less strongly influenced by genetic factors than they are by environmental factors (e.g., diet and disease), while allometry (body proportions) is more strongly influenced by genes than by the environment, the model permits estimation of the environmental and genetic contributions to a child's growth trajectory. The original application of this model entailed fitting it, in a Bayesian framework, to the mean growth trajectories of children from two very different societies: upper‐class Berkeley, California, USA of the 1930s (Tuddenham and Snyder 1954) and contemporary Indigenous Matsigenka communities in Amazonian Peru (with whom authors C.R.M. and J.A.B. work). We interpreted differences in mean trajectories in terms of differential metabolism and allometry between these populations, contextualized, respectively, as differences in diet and disease exposure, and genetic influences on body proportions. Additionally, by using the model to simulate the effects of a healthcare intervention, we demonstrated the potential of such an analysis to contribute to the design of appropriate health support strategies tailored to the needs of a particular population.

Because this new model can be used to investigate both the causes of growth variation in our species, as well as to design tailored healthcare interventions, it is of interest to apply the model to growth data from as many of the world's populations as are interested in contributing to these objectives. Many such populations may be marginalized and/or far from clinical facilities, posing significant logistical challenges for recording height and weight measurements from large numbers of the same children across multiple years. Similarly, in some populations, there is non‐trivial uncertainty about the exact ages of children. In our original study, the US and Matsigenka datasets were both longitudinal, that is, at least some of the same children were measured in different years. Age uncertainty, especially for Matsigenka children, though acknowledged, was not explicitly included in the model fitting strategy. Thus, the original investigation provides little insight into whether the model can be successfully applied to cross‐sectional measures from children of uncertain age.

Applying the model in bioarcheological contexts presents even more acute challenges. Human osteological assemblages may be used to infer growth patterns of past populations, for example, examining how such patterns relate to changes in subsistence and social organization or selection on millennial time scales (Parkinson et al. 2023; Pfeiffer and Harrington 2011), or to disparities in disease and other health challenges among social groups over shorter time periods within a particular region (Lewis 2002; Ribot and Roberts 1996). When assemblages contain sufficient skeletal material from multiple children, a mean child growth trajectory may be estimated for the population, which, if using our new model, can potentially provide insight into the genetic and environmental factors affecting growth. However, such bioarcheological datasets may be small, and necessarily contain a single measure of body size and age at death for each individual, both of which are imperfectly estimated, for example, using bone lengths and features of the dentition/epiphyses, respectively (Baker et al. 2005; Cunningham et al. 2016; Murray et al. 2024; Raxter et al. 2006; Robbins et al. 2010; Ruff et al. 2012; Schaefer et al. 2009; Yapuncich et al. 2018). Thus, it is of interest to know how reliable are estimates of the population mean growth trajectory derived from the application of the model to such data.

Here, we use simulations to investigate the accuracy of model estimates when fit to cross‐sectional (compared to longitudinal) datasets from different numbers of children, knowledge of whose ages is imperfect. Results can help guide analysis of ancient and historical populations and design sampling strategies in contemporary societies whose members desire to participate in such research, but where logistical challenges preclude collecting more than one height and/or weight measurement per child.

## 2 Materials and Methods

### 2.1 Growth Model

The theoretical model presented in Bunce et al. (2025) is adapted from the differential equation model of organismal growth proposed by Pütter (1920), and popularized by von Bertalanffy (1938). In this general model, mass increases when the rate of anabolism (using energy to form chemical bonds between molecules) is greater than the rate of catabolism (breaking chemical bonds to release energy). Because all living cells perform catabolic reactions, catabolism is a function of an organism's mass. In contrast, anabolism is limited by the acquisition of substrate molecules from the environment through an organism's absorbing surface (e.g., intestine). Thus, the instantaneous rate of change in mass is

(1) $$\frac{\mathrm{dm}}{\mathrm{dt}}=\mathrm{Hs}-\mathrm{Km}$$

where *m* is mass, *s* is surface area, H≥0 is synthesized mass (i.e., substrate molecules chemically bonded via anabolism) per unit of an organism's absorbing surface, and K≥0 is destructed mass (i.e., molecules whose chemical bonds are broken via catabolism) per unit of an organism's mass. The shape of the body is stylized as a cylinder with height *h*, radius *r*, and substrate‐absorbing (i.e., intestinal) surface area 2*πrh*. The body changes shape (elongates) as it grows, i.e., *r* and *h* both increase, but *h* increases faster, such that $r=h^{q}$, where 0<q<1 represents the allometric relationship between radius and height. These assumptions yield the following expressions for height and weight, respectively, at total age *t* years since conception:

(2) $$h\left(t\right)={\left[\frac{2H}{K}\left(1-e^{\frac{\mathrm{Kq}}{1+2q}\left(i-t\right)}\right)\right]}^{\frac{1}{q}}$$

(3) $$m\left(t\right)=\pi {\left[\frac{2H}{K}\left(1-e^{\frac{\mathrm{Kq}}{1+2q}\left(i-t\right)}\right)\right]}^{\frac{1}{q}+2}$$

for all t≥i, where i≥0 is the age at which growth is initiated, such that, for growth in early life, i=0 at conception (derivations in Bunce et al. 2025, appendices A and B).

These general expressions apply, in theory, at scales from single cells, to tissues, to bodies. However, in humans, different parts of the body grow at different rates (Eveleth and Tanner 1990, 35, 82) under coordinated cycles that differ in period from days to years (Butler et al. 1990; Michelle Lampl 1993; Lampl et al. 1992; Togo and Togo 1982). To represent the additive effect of such overlapping growth processes on the overall increase in body size, we constructed a composite model (see also Karlberg (1989) and Nierop et al. (2016)) by summing five distinct, yet covarying, growth processes. The choice of five growth process (rather than more or fewer) is designed to balance representation of these intra‐ and inter‐yearly cycles of growth over ontogeny against the temporal density of most available datasets, for example, usually not more than one measurement per individual per year, and often much less (Bunce et al. (2025), appendix C). When fit to empirical data, the ages at which these five component processes tend to have their largest effects on overall growth roughly correspond to commonly distinguished ontogenetic phases: (1) in utero; (2) infancy; (3) early childhood; (4) late childhood; and (5) adolescence. Together, these assumptions yield the following composite growth model for height (Equation 4) and weight (Equation 5) at total age t years since conception:

(4) $$\begin{array}{c} h\left(t\right)=0.012+\sum_{x=1}^{5}\left[t\geq i_{x}\right]{\left[\frac{2H_{x}}{K_{x}}\left(1-e^{\frac{K_{x}q_{x}}{1+2q_{x}}\left(i_{x}-t\right)}\right)\right]}^{\frac{1}{q_{x}}} \end{array}$$

(5) $$\begin{array}{c} m\left(t\right)=1.02\cdot 10^{-6}+\sum_{x=1}^{5}\left[t\geq i_{x}\right]\pi {\left[\frac{2H_{x}}{K_{x}}\left(1-e^{\frac{K_{x}q_{x}}{1+2q_{x}}\left(i_{x}-t\right)}\right)\right]}^{\frac{1}{q_{x}}+2} \end{array}$$

such that $i_{1}=0$ represents conception, and $\left[t\geq i_{x}\right]$ is Iverson notation (Knuth 1992), taking the value of one if the condition within brackets evaluates to True, and is zero otherwise The first terms in Equations (4) and (5) are the height (diameter, cm) and weight (g), respectively, of a human ovum.

### 2.2 Simulation

Our goal is to fit the model to a range of differently‐structured datasets that correspond to a range of different data collection scenarios potentially encountered in the real world. Importantly, to assess the accuracy of growth trajectories estimated by the model when fit to each dataset, the true growth trajectory in each case must also be known. If all such data collection scenarios of interest could be represented as subsets of an existing large empirical dataset, then the model could potentially be tested by comparing trajectories estimated from these subsets against the “true” trajectory estimated from the large complete dataset. In the present study, our interests lie in a particular set of scenarios, some of which differ in sample size and/or measurement schedule from any existing empirical dataset that we know of. For instance, we are interested in comparing the performance of the model when fit to a cross‐sectional dataset derived from 100 individuals (ages 0–25) from a population with an average growth trajectory very different from that of the US population (Figure 3), against a longitudinal dataset from this same population where 20 individuals (ages 0–25) were each measured five times at two‐year intervals (Supporting Information Appendix D). The empirical data we have access to come from a Matsigenka population of 196 girls between one and 24 years old, each measured a maximum of three, and an average of 1.3, times (Bunce et al. 2025). While the cross‐sectional data collection scenario above could be adequately represented as a subset of the complete Matsigenka dataset, we are unaware of a publicly‐available dataset from a non‐EuroAmerican population of which the second data collection scenario above would be a subset.

To address this problem, we use properties of empirical datasets to create a broad range of simulated data structures of interest. We focus on a hypothetical situation in which a researcher has very little a priori information about the mean growth trajectory (her target of inference) of a focal population. Her best guess is that the mean trajectory is broadly similar to that previously estimated for US children, which we represent as the “prior” trajectory (Bunce et al. (2025), used below in Bayesian model fitting). To challenge our model, we want this prior best guess to be a fairly poor one. Thus, we simulate datasets from a focal population whose mean growth trajectory matches that of the Matsigenka (defined by the set of H's, K's, q's, and i's in Equations 4 and 5), who tend to be considerably shorter than people from the US (Bunce et al. 2025). We define this Matsigenka mean growth trajectory as the “true” mean trajectory for the simulated datasets, and this is what the hypothetical researcher is attempting to estimate using measurements from individuals. However, the growth trajectories of individual Matsigenka do not exactly match their population's mean trajectory. Thus, in order to realistically simulate measurements from individuals, we must know the inter‐individual variance around the mean trajectory. Our empirical dataset from the Matsigenka is temporally sparse, which makes it difficult to accurately estimate individual growth trajectories, and therefore inter‐individual variance (Bunce et al. 2025). However, under the assumption that variance in growth among Matsigenka children is similar to that of US children, we simulate measurement data using inter‐individual variance derived from analysis of a temporally‐dense dataset from 70 US girls, each measured 30 times, on average, at regular intervals between birth and Age 21 (Bunce et al. 2025; Tuddenham and Snyder 1954). Similarly, covariance among model parameters across the five growth processes within each individual, which is also difficult to estimate with temporally‐sparse data, is derived from the US dataset. This strategy allows us to simulate data from any number of individuals, measured any number of times, from a population whose mean growth trajectory matches that of the Matsigenka, and whose inter‐individual variance around this mean trajectory, as well as individual‐level covariance, matches that of US children.

**FIGURE 1:**
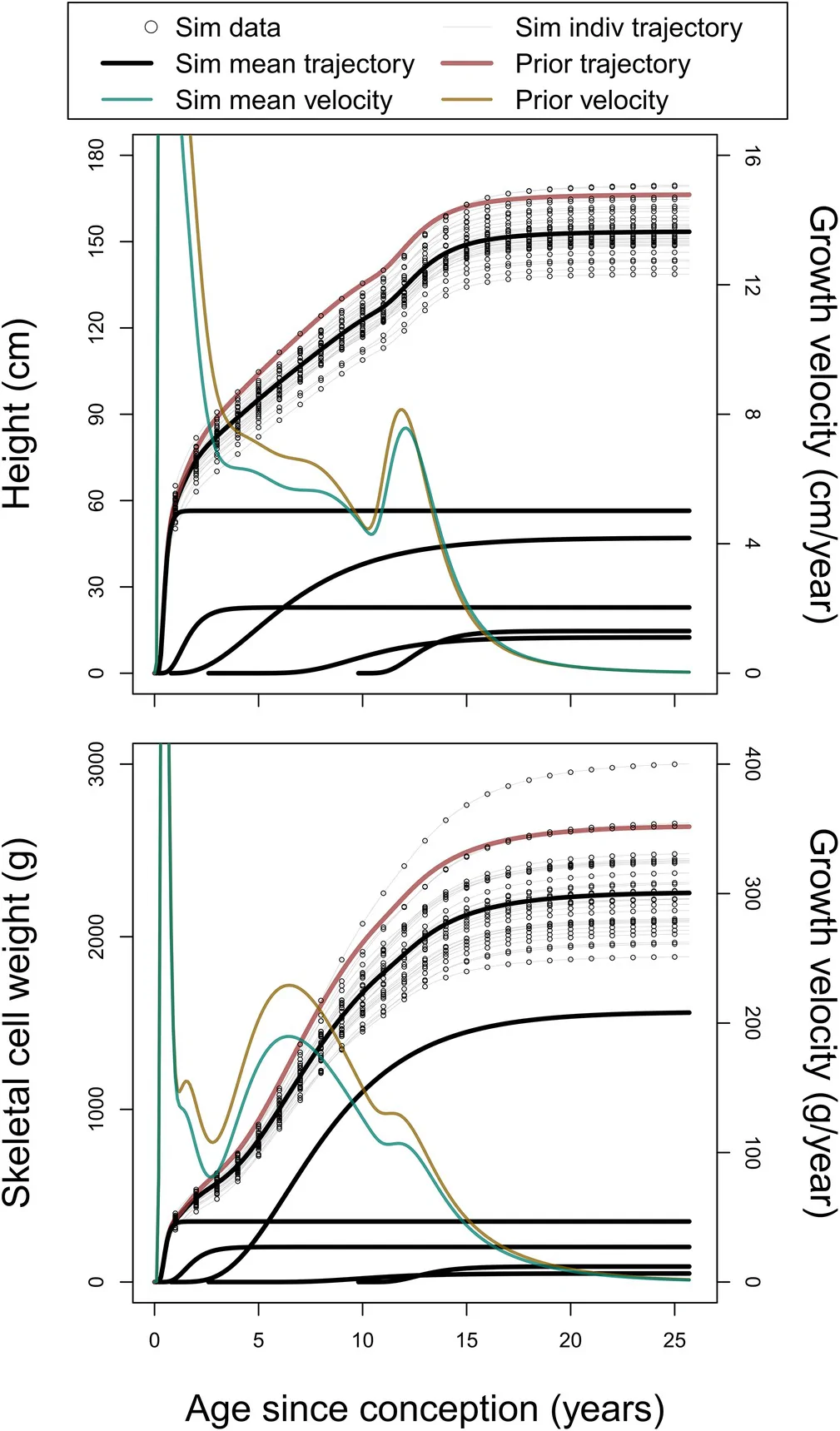
Height and weight data simulated for 30 individuals measured every year from birth to age 25 using Equations (4) and (5). Parameter values defining the simulated mean cumulative growth trajectory and five component processes are derived from Matsigenka girls, while individual‐level variance around the mean trajectory is derived from US girls. The five component processes in the lower half of each plot roughly correspond (from left to right) to growth in: (1) utero; (2) infancy; (3) early childhood; (4) late childhood; and (5) adolescence. The prior growth trajectory (red) used to fit the model to the simulated data is derived from the mean trajectory of US girls. Corresponding mean cumulative velocity trajectories in green and orange are decreasing (after age 14) from left to right. Bunce et al. (2025) explains the complications of fitting (and simulating) weight data using the model.

For each simulated individual within a dataset, a single random age (cross‐sectional datasets) or a set of ages (longitudinal datasets) is chosen between one and 25 years since conception. Height and weight at each age are calculated using Equations 4 and 5, together with that individual's unique set of offsets (random effects) from the population mean height and weight, drawn from a multivariate Normal distribution with a variance–covariance matrix derived from the US children. Figure 1 shows an example simulated longitudinal dataset for height and weight.

**FIGURE 2:**
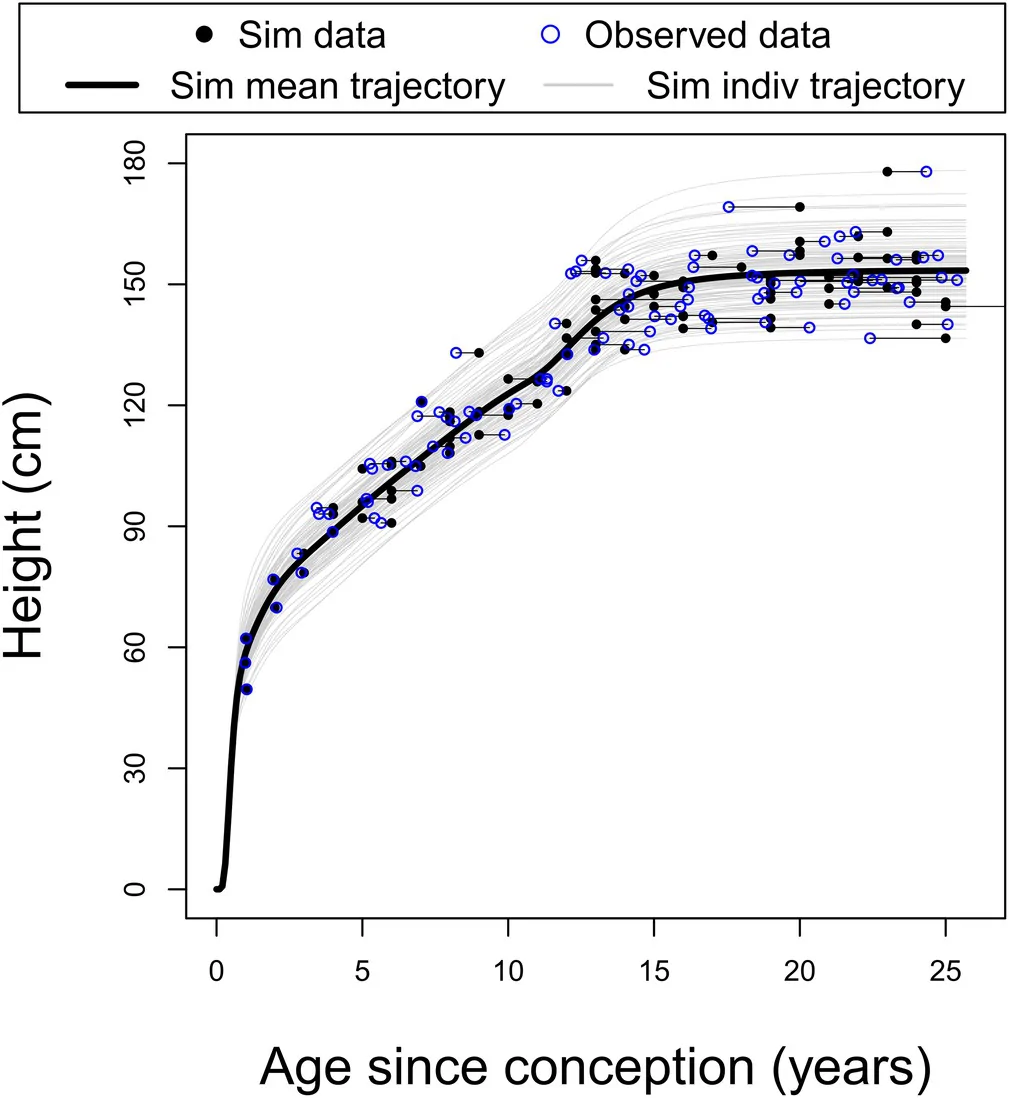
Simulated cross‐sectional height data for 100 individuals (black points), where the thick black line is the true population‐mean growth trajectory and gray lines are true individual trajectories. Horizontal lines connect each point to the potentially‐inaccurate “observed” age for that individual (blue circles), which incorporates uncertainty in the age recorded for each child during height measurement. Note that uncertainty tends to increase with age.

Uncertainty in age manifests as uncertainty in each individual's date of birth, and is modeled as

(6) $$\begin{array}{c} y∼\text{Normal}\left(y_{T},\sigma_{\varepsilon }\right) \end{array}$$

where $y_{T}$ is the (potentially unobserved) true date of birth (in years), and y is the potentially inaccurate date of birth recorded for an individual when they are measured for the first time. The standard deviation, $\sigma_{\varepsilon }=a\cdot \varepsilon$, is the product of true age (a, calculated from $y_{T}$ and the date of measurement) and the uncertainty factor ε=0.1. Note that $\sigma_{\varepsilon }$ increases with age, representing our experience as fieldworkers that there is often more uncertainty about the (relatively distant) date of birth of someone measured for the first time as an older adult than there is about the (more recent) birthdate recorded during measurement of a young child. The observed, potentially inaccurate, total age since conception, t, is calculated as $\left(y-0.75\right)$, subtracted from the date of measurement. Figure 2 illustrates age uncertainty in an example simulated cross‐sectional dataset for height.

### 2.3 Fitting the Model

Our objective is to fit the growth model to the simulated data in a Bayesian framework, using priors that define a mean growth trajectory very different from that used to simulate the data, in order to evaluate how close the resulting posterior mean trajectory is to the true mean trajectory for the simulated dataset. This provides insight into the degree of confidence we can have in estimates of the model fit to cross‐sectional (and other types of) datasets.

Height $h_{\mathrm{jt}}$ and weight $m_{\mathrm{jt}}$ of person j at observed total age t years since conception are estimated as:

(7) $$\begin{array}{c} \ln\left(h_{\mathrm{jt}}\right)∼\text{Normal}\left[\ln\left(\eta_{\mathrm{jt}}\right),\sigma_{\eta }\right] \end{array}$$

(8) $$\begin{array}{c} \ln\left(m_{\mathrm{jt}}\right)∼\text{Normal}\left[\ln\left(\mu_{\mathrm{jt}}\right),\sigma_{\mu }\right] \end{array}$$

where $\eta_{\mathrm{jt}}$ and $\mu_{\mathrm{jt}}$ are Equations (4) and (5), respectively, which, when each parameter is indexed by j, represent height and weight trajectories of individual j in the five growth processes. To represent the fact that observed heights and weights can never be negative, we take the log of both the observations and the mean of the Normal likelihood. The standard deviations $\sigma_{\eta }$ and $\sigma_{\mu }$ represent measurement error, on the log scale, around individual $j^{’}$s actual height and weight, respectively, at observed total age t. To aid convergence, the model, with covarying group‐level and individual‐level random effects, is fit to a combined dataset comprising the cross‐sectional simulated data, together with temporally‐dense longitudinal data from 70 girls in the US dataset. See Cole et al. (2016) for an analogous strategy fitting sparse and dense datasets together. Priors on model parameters are derived from mean posteriors of the model previously fit to these longitudinal data from the US children (Bunce et al. 2025). Models were fit in R (R Core Team 2022) and Stan (Stan Development Team 2022) using the cmdstanr package (Gabry and Cesnovar 2021). Complete model structure, priors, and additional details are provided in Supporting Information Appendix A. Data simulation and analysis scripts are provided at https://github.com/jabunce/bunce‐revilla‐fernandez‐2026‐age_uncert.

The model was fit to simulated cross‐sectional datasets representing 10, 100, and 200 individuals. Five datasets of each sample size were independently simulated, and the model was fit to each one in order to represent stochastic variability in the particular individuals who become incorporated into empirical datasets. Accuracy of model posterior parameter estimates was assessed by computing the 90% highest posterior density interval (HPDI: McElreath (2020)) for each estimate. If this interval included the true value of a parameter, then the model estimate was deemed accurate.

## 3 Results

**FIGURE 3:**
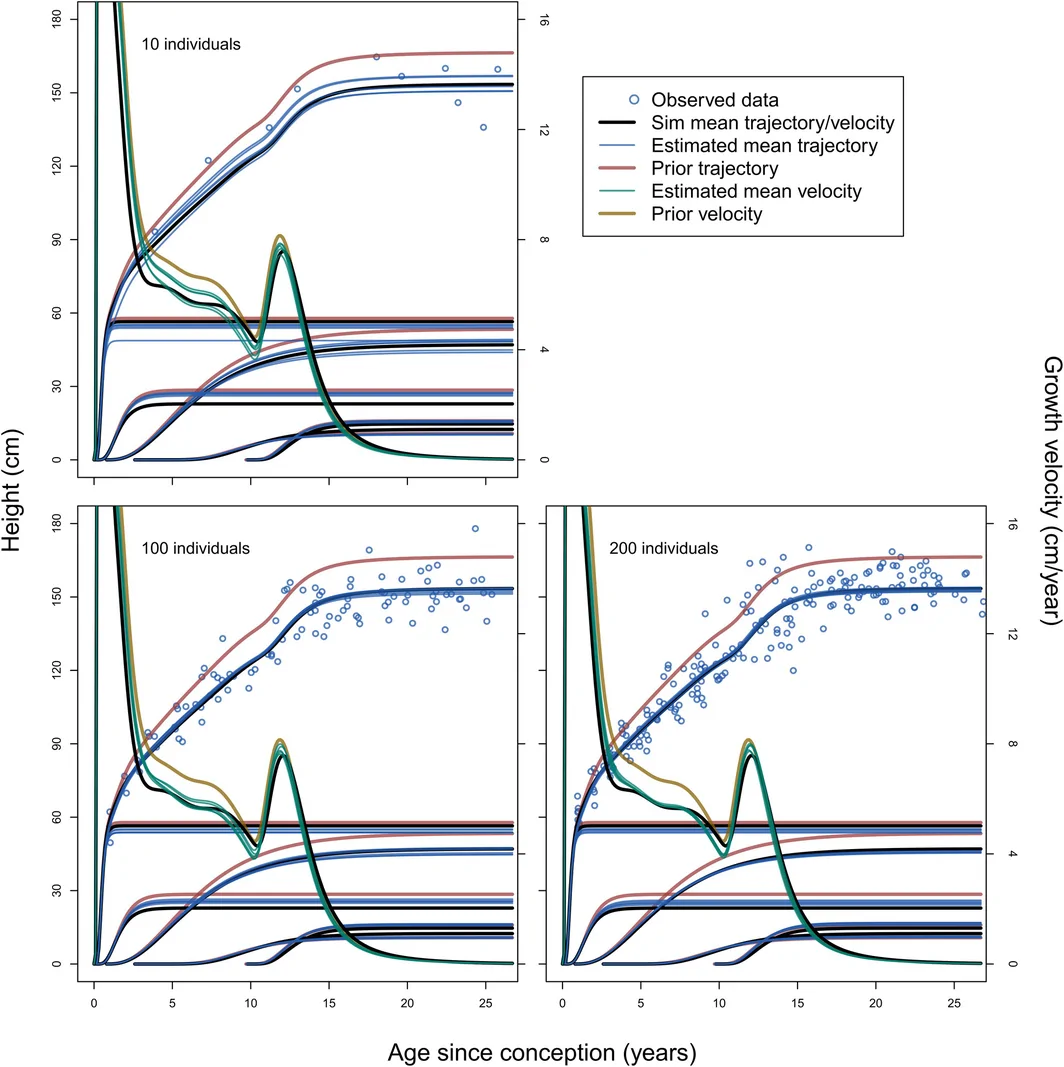
Mean posterior cumulative population‐mean height growth trajectories and component processes (blue lines) estimated from five independently‐simulated cross‐sectional datasets varying in size from 10 to 200 individuals. Data points in each plot come from one of the five simulations for a dataset of a given size, incorporating age uncertainty (Figure 2). Black lines increasing from left to right are, respectively, the true simulated mean growth trajectory and component processes. Red lines are the respective priors used to fit the model to each dataset. Corresponding mean cumulative velocity trajectories in black, orange, and green are decreasing (after age 14) from left to right. Note that the five blue and five green lines (from the five simulations) in each plot are often too close together to clearly distinguish one from another. Also note that, with small datasets, posterior estimates are more heavily influenced by (more closely match) the prior.

We focus our analysis on trajectories for growth in height, as model estimates for weight growth are expected, on theoretical grounds, to be less accurate (Bunce et al. 2025). Figure 3 shows that, for cross‐sectional datasets, the accuracy of model estimates of the population mean growth trajectory increases as the number of measured individuals increases. With more data, posterior estimates are less influenced by the priors, and track closer to the true mean trajectory. Before birth, where there are no simulated measurements, it is very difficult to accurately estimate the pattern of growth. Furthermore, different additive combinations of the first two growth processes yield similar cumulative trajectories, which are difficult for the model to distinguish when data are absent or sparse. During the pubertal growth spurt, when growth velocity is high, and there is considerable inter‐individual variation in magnitude and onset, accurate estimation of the mean trajectory is also less accurate, as can be seen in the fifth growth process.

**FIGURE 4:**
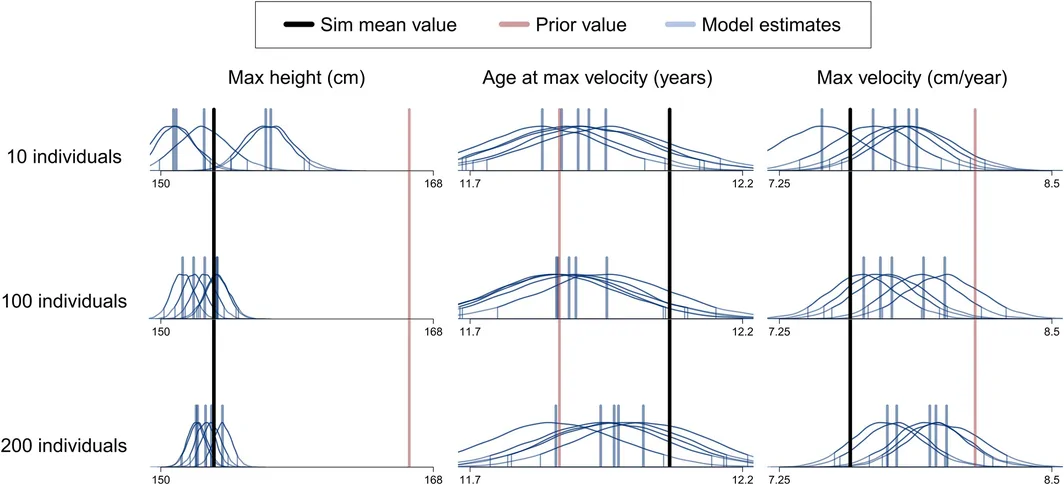
Three characteristics of the population‐mean height growth trajectories illustrated in Figure 3: maximum achieved height (left column), age since conception at which the maximum growth velocity during puberty is observed (center column), and maximum growth velocity during puberty (right column). Five posterior distributions (blue) come from fitting the model to five independently‐simulated cross‐sectional datasets of between 10 and 200 individuals (rows). Vertical blue lines are means of each posterior distribution, and marked distribution tails enclose 90% highest posterior density intervals (HPDI: McElreath (2020)). HPDIs that overlap the actual population‐mean value of the simulated datasets (black vertical lines) indicate accurate, though not necessarily precise, posterior estimates.

Figure 4 incorporates uncertainty in the posterior estimates to compute descriptive characteristics of the growth trajectories. Accuracy of estimates of mean maximum attained height increase with the number of people measured. However, the accuracy of mean maximum growth velocity at puberty, and the mean age at which it is obtained, are not substantially improved by increasing the number of cross‐sectional measurements from 10 to 200. This highlights the difficulty of estimating the mean pubertal growth trajectory using cross‐sectional data.

**FIGURE 5:**
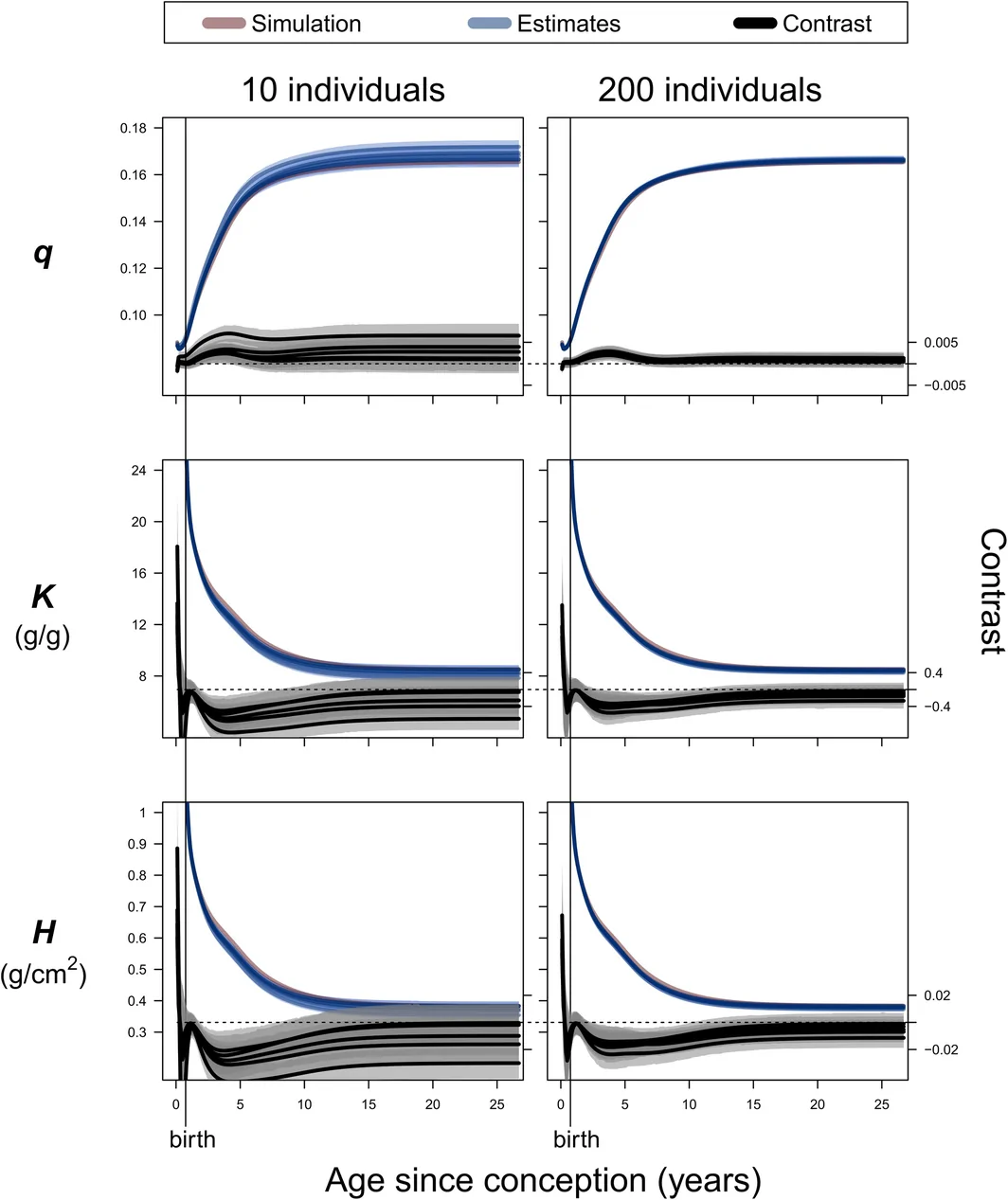
Estimates of mean parameter values by age. Blue lines are means and shaded areas are 90% HPDI for posterior distributions of weighted sums of population‐mean values of model parameters q (allometry), K (catabolism), and H (anabolism) across the five component growth processes of the model in Equations (4) and (5) (Bunce et al. 2025), estimated for five independently‐simulated cross‐sectional datasets of 10 (left column) and 200 (right column) individuals. The five estimated parameter trajectories and respective HPDIs in each plot are often too narrow to be distinguished one from another. Red lines in each plot are calculated from the actual parameter values used in the simulations. The 90% HPDIs of the contrasts (differences) between each of the five posterior estimates and the actual simulation value are shown in gray, with means in black. HPDIs of contrasts that overlap zero (dotted horizontal lines) indicate an accurate (though not necessarily precise) estimate of a particular parameter at a particular age. Note that the accuracy of parameter estimates for K and H tend to improve after age 15, especially for cross‐sectional datasets of 200 individuals.

A strength of the growth model in Equations (2) and (3) is that parameters are biologically interpretable, such that q represents allometry, K and H represent metabolic rates, and i represents the age at which a growth process is initiated. Posterior estimates for all parameters are shown in Supporting Information Appendix Figures A.1 and A.2. Following Bunce et al. (2025), population‐mean parameter estimates for q, K, and H may be combined (via weighted sums) for each age over the entire period of growth. Figure 5 shows that accuracy of such estimates improves with a larger sample of measured people. However, even with a cross‐sectional sample of 200 people, population mean parameter estimates may notably deviate from the true values before age five (q) or 15 (K and H) years since conception.

## 4 Discussion

We have shown that the new composite growth model of Bunce et al. (2025) can be used to estimate a population mean growth trajectory using cross‐sectional data from children of uncertain age. As the number of measured individuals increases, the accuracy of the estimate of the mean maximum achieved height improves (Figures 3 and 4). However, there is much less improvement in model estimates of features of the mean trajectory during the pubertal growth spurt, even with measurements from 200 children (Figure 4). This places important limits on interpretation when comparing a mean growth trajectory from one population, estimated with this model using cross‐sectional data, against a growth standard, or against the mean trajectory of a different population. Because, to our knowledge, this is the first analysis of its kind for a human growth model, we focus our discussion on topics potentially of interest to researchers working with datasets having different types and degrees of limitation: We compare the effects on accuracy of explicitly incorporating age uncertainty into the model, excluding measures of weight, and adopting several strategies of targeted longitudinal data collection.

### 4.1 Modeling Age Uncertainty

In the simulated dataset, uncertainty in age is represented as a random offset from the true age, with the magnitude of the offset increasing with the magnitude of age (i.e., more age uncertainty for older individuals: Figure 2). Because these age offsets are random with respect to true age, they should have no effect, on average, on estimation of the population mean growth trajectory. We test this intuition by explicitly incorporating age uncertainty into the model. This entails estimating the year of conception for each individual using a Gaussian likelihood centered on an individual's recorded year of conception (recorded year of birth—0.75), and standard deviation scaled by recorded age at the time of measurement (analogous to how age uncertainty is simulated). As shown in Supporting Information Appendix B, this strategy results in a posterior distribution for the age of each individual, the mean of which tends to be shifted such that the data point falls closer to the estimated mean growth trajectory of the population (Supporting Information Appendix Figure A.3). However, comparing Figures 3, 4, 5 with Supporting Information Appendix Figures A.4, A.5, and A.8, illustrates that estimating ages in this way has little effect on the accuracy of the estimated mean growth trajectory, which is our target of inference. Interestingly, Koster et al. (2020) observed a similar (absence of) effect when modeling age uncertainty in the context of a theory of skill‐development. We conclude that, when fitting this growth model to cross‐sectional data from individuals of uncertain age, where such uncertainty is plausibly random with respect to the actual age, modeling this age uncertainty, while justified, may yield little practical benefit for estimation of the mean growth trajectory.

### 4.2 Fitting the Model Without Weight

The composite growth model is designed to simultaneously fit height and weight measurements from the same individuals, as this tends to result in more consistent parameter estimates with longitudinal data (Bunce et al. (2025), appendix D.1.3). It is of interest to know how the absence of weight information affects estimation of the population mean growth trajectory when fitting the model to cross‐sectional data. This is accomplished by using the model to impute weights in the simulated dataset as if they were missing data (McElreath 2020), as detailed in Supporting Information Appendix C. Comparing Figures 3, 4, and 5 with Supporting Information Appendix Figures A.4, A.5, and A.8 illustrates that imputing cross‐sectional weights has little effect on estimation of the population mean growth trajectory. We conclude that little accuracy is lost when fitting this model to cross‐sectional height data in the absence of weight measures.

### 4.3 Cross‐Sectional Versus Longitudinal Measures

The composite growth model was originally designed to fit longitudinal, rather than cross‐sectional, growth data. Thus, it is of interest to compare the accuracy of estimates when the model is fit to the same number of measurements obtained using cross‐sectional versus longitudinal data collection strategies (Supporting Information Appendix D). Supporting Information Appendix Figure A.4 shows the estimated mean growth trajectory derived from 50 individuals, each measured twice, with an interval of 2 years between measurements. Supporting Information Appendix Figure A.9 shows the trajectory derived from 10 individuals, each measured 10 times at one‐year intervals, as well as from 20 individuals, each measured five times at two‐year intervals. As each of these longitudinal strategies results in a total of 100 data points, their estimates can be compared against the growth trajectory in Figure 3 derived from 100 individuals measured once. Comparing Figure 4 with Supporting Information Appendix Figures A.5 and A.10, it is apparent that estimation of the age of maximum growth velocity during puberty improves with a longitudinal study design, particularly one entailing 20 individuals measured five times at 2‐year intervals. Accuracy of posterior estimates of maximum achieved height and maximum growth velocity during puberty are comparable between sampling designs. We conclude that the same amount of data, collected using a longitudinal rather than cross‐sectional design, tends to yield slightly more accurate estimates of the growth trajectory, especially during puberty.

### 4.4 Metabolic and Allometric Parameters

Interpreting metabolic parameters K and H, and the allometric parameter q, for each of the five individual growth processes is known to be difficult, as these processes overlap in time. Because height and weight measurements represent the body as a whole, it is not possible to collect post‐natal measurements that represent only a single growth process. Thus, at a given age, there may be different combinations of growth processes (with distinct values of K, H, and q) that sum to the same, or very similar, overall height and weight. In the absence of process‐specific data, it is difficult to distinguish among these possibilities without very strong, (in this case) arbitrary, priors. This ambiguity contributes to the inaccuracy of model estimates of process‐specific parameters (especially K and H in later growth phases), even with extensive longitudinal measurements from 30 individuals (Supporting Information Appendix Figures A.1, A.6, and A.11). Estimates of process‐initiation parameters i, which are not summed, tend to be more accurate (Supporting Information Appendix Figures A.2, A.7, and A.12).

Bunce et al. (2025), describe a physiologically‐informed procedure to calculate a weighted sum of posterior estimates of metabolic and allometric parameters across the five growth processes, which results in interpretable overall values of K, H, and q at each age during ontogeny. Figure 5 shows that these composite parameter estimates, derived from cross‐sectional data, tend to be relatively inaccurate at younger ages. However, Supporting Information Appendix Figures A.8 and A.13 demonstrate incremental improvements in accuracy with longitudinal datasets, such that reasonable uncertainty bounds on parameter estimates derived from a model fit to 25 yearly measures from each of 30 individuals tend to include the actual parameter values over all of ontogeny (Supporting Information Appendix Figure A.13). We conclude that caution should be exercised when interpreting estimates of composite metabolic and allometric parameters, especially at younger ages, derived from a model fit to cross‐sectional data.

### 4.5 Application and Limitations

The growth model presented here is designed to be fit to height, weight, and age data from one or more populations. The resulting estimates of model parameters can be used to visualize population mean growth trajectories, and to compare the relative contributions of metabolic rates (*K* and *H*) and allometry (*q*) to population‐level differences in these trajectories. Often, however, researchers have additional information about the study population(s) and/or the measurement process that can be included in the statistical analysis to provide more accurate parameter estimates. Below we provide several examples of how to do this, followed by limitations of our approach.

In the model‐fitting analyses above, we assume there is uncertainty in both height and age measurements, and that measurement errors are randomly distributed about the true values. We assume that uncertainty in age increases with age by a constant factor (Supporting Information Appendix B, equation A.32), as does uncertainty in height (Bunce et al. (2025), appendix D.2 pp. 33). Furthermore, we assume that all measured individuals are subject to these same uncertainty properties. However, it may be the case, particularly with cross‐sectional bioarcheological data, that the degree of uncertainty (e.g., measurement error) in height is known to differ between two individuals measured as having the same height (e.g., if individuals differ in the degree of osteological preservation). This additional uncertainty information can be readily incorporated into the statistical model by replacing the standard deviation in height ($\sigma_{\eta }$) in Equation (7) with a vector of *J* unique standard deviations ($\sigma_{\mathrm{\eta j}}$), one for each individual *j*. These standard deviations can be chosen such that they encode the additional individual‐specific uncertainty information, as explained in Supporting Information Appendix A.1.

Similarly, it may be the case that uncertainty in age is known to differ between two individuals recorded as having the same age. In that case, the standard deviation of the estimated year of conception ($\sigma_{\varepsilon }$ in Supporting Information Appendix A, Equation A.31) can be replaced with a vector of *J* unique standard deviations ($\sigma_{\mathrm{\varepsilon j}}$), analogous to the strategy for height above (though without transformation onto the log scale: Supporting Information Appendix B.1). Additionally, if it is known that error in age measurement is non‐randomly distributed about the true age (e.g., there is systematic over‐ or under‐estimation), then the symmetric Gaussian prior on the year of conception (Appendix A, Equation A.31) can be replaced with an asymmetrical prior that better reflects how probability should be distributed on either side of the recorded year of conception.

In the statistical analyses presented here, we assume that the sex of all individuals is known to be female, which justifies our use of priors derived from US girls (Appendix A, Equations A.15–A.18). Following Bunce et al. (2025), we recommend that separate models be fit to data from males and females, as growth trajectories tend to differ, on average, by sex. However, in bioarcheological contexts, the sex of young individuals may be difficult to determine. Although further investigation is needed, our intuition is that including very young individuals of mixed sex within an overall sex‐specific dataset of heights and weights is unlikely to strongly distort model estimates of the population‐mean growth trajectory for the focal sex. For instance, Bunce et al. (2025) Figure 2, suggests that height trajectories of male and female children (both US and Matsigenka) begin to notably diverge only after approximately 7 years since conception, when the fourth and fifth growth processes (late childhood and adolescence) begin to affect overall height. Thus, we hypothesize that including individuals of unknown sex who are younger than this age in a dataset where the remaining individuals are known to be female, is unlikely to strongly distort estimation of the mean female growth trajectory (and analogously for a male dataset). This conjecture remains to be explored, particularly in other populations.

The data contexts encountered by many researchers are not adequately represented by the analyses presented here. However, in some cases we believe that reasonable conjectures can be made (subject to future testing). For instance, we expect that modestly increasing the degree of age uncertainty (ε>0.1) and the degree of height and weight uncertainty ($\sigma_{\eta }$ and $\sigma_{\mu }$, Appendix A) will have little effect on model estimates of the population mean growth trajectory, as long as such uncertainty is random with respect to the true values (see section Modeling age uncertainty). Similarly, we expect our results to hold for datasets simulated to represent males instead of females, as long as the priors on model parameters are also derived from male populations (e.g., male parameter estimates reported in Bunce et al. (2025)). For other scenarios, targeted modeling exercises are required. For instance, if a researcher anticipates a cross‐sectional sample of a size between 10 and 100 individuals, then datasets of the anticipated size should be simulated in order to predict model accuracy for the specific sample. Similarly, additional simulations would be required to assess model accuracy if age uncertainty is non‐random (i.e., biased), or if a dataset mixing cross‐sectional measures from some individuals and longitudinal measures from others is anticipated. Instructions in Supporting Information Appendix A.1, along with open‐access simulation and analysis scripts are provided to allow other researchers to tailor these simulations to their needs.

## 5 Conclusions

Results of this study suggest that the composite growth model of Bunce et al. (2025) is a useful tool to estimate population mean growth trajectories, even for populations of children whose growth differs markedly from that of typical US children, and from whom only single measures of height may be collected from individuals whose ages are (randomly) uncertain. In such a context, fitting the model to measurements of 100 children of a given sex, with ages spread relatively evenly over ontogeny, is likely to provide an estimate of the mean growth trajectory that is sufficiently accurate for broad comparisons between populations (Figure 3 and Supporting Information Appendix Figure A.4). In this example, such a dataset would likely result in estimates of mean maximum achieved height, mean age at maximum pubertal velocity, and maximum pubertal velocity within, respectively, 5 cm, 0.5 years, and 1 cm/year of the true values (Figure 4 and Supporting Information Appendix Figure A.5). Accuracy may increase if the study population exhibits a growth pattern similar to that of the population from which the prior is derived, and may decrease if the prior is derived from a population with a very different growth pattern. In the future, as child growth from more populations around the world is analyzed using this model, a more diverse range of growth trajectories will become available from which to make an informed choice of an appropriate prior (e.g., based on similar ecology or genetic ancestry) for analysis of additional populations (Decrausaz and Cameron 2022).

Results above suggest that the model may be particularly useful for analysis of the population mean growth trajectories of historical populations, where bioarcheological data are necessarily cross‐sectional, age is estimated with uncertainty, and height may be more easily and directly estimated from bone length (Murray et al. 2024; Raxter et al. 2006) than weight, the estimation of which may be problematic as it often depends on estimated stature (Ruff et al. 2012; Yapuncich et al. 2018) and on assumptions about unpreserved soft tissue (Robbins et al. 2010). We have shown above that, in such contexts, weight estimates may be excluded from analysis with little loss of accuracy in estimates of the population mean height trajectory. Of special interest are archaeological contexts where large numbers of presumably healthy children died suddenly (e.g., child sacrifice in ancient South American societies: Prieto et al. (2024) and Prieto et al. (2019)). Such osteological assemblages, analyzed with this model, could provide an important window into patterns of healthy growth in ancient and historical populations, and a better understanding of how and why these patterns may differ from those of contemporary populations (Decrausaz and Cameron 2022).

Finally, while this analysis shows that important insights into the population mean growth trajectory can be obtained from cross‐sectional data, estimates of growth characteristics are often considerably less accurate than those derived from longitudinal datasets, especially with regard to the pubertal growth spurt, and for interpretation of metabolic and allometric parameters. Thus, a research question related to these aspects of growth in under‐studied, remote, and/or marginalized societies may necessitate investing additional effort, in collaboration with participants, to record longitudinal measures of children's height and weight.

## Author Contributions

**John A. Bunce:** conceptualization, investigation, funding acquisition, writing – original draft, methodology, validation, visualization, writing – review and editing, software, formal analysis, project administration, data curation, supervision, resources. **Caissa Revilla‐Minaya:** conceptualization, investigation, funding acquisition, methodology, writing – review and editing, project administration, supervision, data curation, resources, validation. **Catalina I. Fernández:** conceptualization, data curation, investigation, methodology, resources, validation, writing – review and editing.

## Funding

Funding was provided by the Max Planck Society and the American Museum of Natural History.

## Ethics Statement

This study uses only simulated and previously published data.

## Conflicts of Interest

The authors declare no conflicts of interest.

## Supporting information


**Appendix A.** Base statistical model.
**Appendix B**. Modeling age uncertainty.
**Appendix C**. Fitting the model to height alone.
**Appendix D**. Fitting the model to longitudinal data.
**Figure A.1**. Posterior estimates of population‐mean parameter values for each of the five growth processes in the composite growth model (Bunce et al. 2025, appendix C), when fit to simulated cross‐sectional datasets from 10 (left column), 100 (center column), and 200 (right column) individuals. Five posterior distributions (blue) come from fitting the model to five independently‐simulated cross‐sectional datasets for each sample size. Vertical blue lines are means of each posterior distribution, and marked distribution tails enclose 90% highest posterior density intervals (HPDIs). HPDIs that overlap the actual population‐mean value of the simulated datasets (red vertical lines) indicate accurate, though not necessarily precise, posterior estimates.
**Figure A.2**. Posterior estimates of population‐mean values of initiation parameters, *i*, for each of the five growth processes (columns), when fit to simulated cross‐sectional datasets from 10 (top row), 100 (center row), and 200 (bottom row) individuals. Five posterior distributions (blue) come from fitting the model to five independently‐simulated cross‐sectional datasets for each sample size. Vertical blue lines are means of each posterior distribution, and marked distribution tails enclose 90% HPDIs. HPDIs that overlap the actual population‐mean value of the simulated datasets (red vertical lines) indicate accurate, though not necessarily precise, posterior estimates.
**Figure A.3**. Simulated cross‐sectional height data for 100 individuals (black points), where thick black lines are the true population‐mean growth trajectories and gray lines are individual trajectories. Horizontal lines connect each point to the potentially‐inaccurate “observed” data for that individual (blue circles), which incorporate uncertainty in the age recorded for each child during height measurement. Horizontal lines also connect each observed data point to the posterior mean location of each data point estimated by the model incorporating age uncertainty (red circles). Note that model estimates of data points tend to be shifted toward the population mean trajectory relative to the observed data. See also Figure 1 in the main text.
**Figure A.4**. Mean posterior cumulative population‐mean height growth trajectories and component functions (blue lines) estimated using a model incorporating age uncertainty and applied to five independently‐simulated datasets for each of three measurement strategies: single height and weight measures from 100 individuals (upper left), single height (but not weight) measures from 100 individuals (lower left), and height and weight measures from 50 individuals, each measured twice at an interval of 2 years (lower right). Data points (observed rather than true: Figure 2) in each plot come from one of the five simulations for a dataset of a given type. Plot interpretation is analogous to Figure 3 in the main text.
**Figure A.5**. Three characteristics of the population‐mean height growth trajectories illustrated in Appendix Figure A.4: maximum achieved height (left column), age since conception at which the maximum growth velocity during puberty is observed (center column), and maximum growth velocity during puberty (right column). Five posterior distributions (blue) come from fitting the composite model incorporating age uncertainty to five independently‐simulated datasets comprising single height and weight measures from 100 individuals (top row), single height (but not weight) measures from 100 individuals (center row), and height and weight measures from 50 individuals, each measured twice at an interval of 2 years (bottom row). Plot interpretation is analogous to Figure 4 in the main text.
**Figure A.6**. Posterior estimates of population‐mean parameter values for each of the five growth processes in the composite model incorporating age uncertainty, when fit to simulated datasets comprising single height and weight measures from 100 individuals (left column), single height (but not weight) measures from 100 individuals (center column), and height and weight measures from 50 individuals, each measured twice at an interval of 2 years (right column). Plot interpretation is analogous to Appendix Figure A.1.
**Figure A.7**. Posterior estimates of population‐mean values of initiation parameters, *i*, for each of the five growth processes (columns), when fit to simulated datasets comprising single height and weight measures from 100 individuals (upper row), single height (but not weight) measures from 100 individuals (middle row), and height and weight measures from 50 individuals, each measured twice at an interval of 2 years (bottom row). Plot interpretation is analogous to Appendix Figure A.2.
**Figure A.8**. Estimates of mean parameter values by age. Blue lines are means and shaded areas are 90% HPDI for posterior distributions of weighted sums of population‐mean values of model parameters *q* (allometry), *K* (catabolism), and *H* (anabolism) across the five component growth processes of the model incorporating age uncertainty estimated for five independently‐simulated datasets comprising single height (but not weight) measures from 100 individuals (left column), and height and weight measures from 50 individuals, each measured twice at an interval of 2 years (right column). Plot interpretation is analogous to Figure 5 in the main text.
**Figure A.9**. Mean posterior cumulative population‐mean height growth trajectories and component functions (blue lines) estimated using a model (without incorporating age uncertainty) applied to five independently‐simulated datasets for each of three measurement strategies: height and weight measures from 10 individuals measured yearly for 10 years (upper left), 20 individuals measured five times at two‐year intervals (lower left), and 30 individuals measured yearly for 25 years (lower right). Data points (observed rather than true: Figure 2) in each plot come from one of the five simulations for a dataset of a given type. Plot interpretation is analogous to Figure 3 in the main text.
**Figure A.10**. Three characteristics of the population‐mean height growth trajectories illustrated in Appendix Figure A.9: maximum achieved height (left column), age since conception at which the maximum growth velocity during puberty is observed (center column), and maximum growth velocity during puberty (right column). Five posterior distributions (blue) come from fitting the composite model (without incorporating age uncertainty) to five independently‐simulated datasets comprising height and weight measures from 10 individuals measured yearly for 10 years (top row), 20 individuals measured five times at two‐year intervals (middle row), and 30 individuals measured yearly for 25 years (bottom row). Plot interpretation is analogous to Figure 4 in the main text.
**Figure A.11**. Posterior estimates of population‐mean parameter values for each of the five growth processes in the composite model (without incorporating age uncertainty) when fit to simulated datasets comprising height and weight measures from 10 individuals measured yearly for 10 years (left column), 20 individuals measured five times at two‐year intervals (middle column), and 30 individuals measured yearly for 25 years (right column). Plot interpretation is analogous to Appendix Figure A.1.
**Figure A.12**. Posterior estimates of population‐mean values of initiation parameters, *i*, for each of the five growth processes (columns), when the model (without incorporating age uncertainty) is fit to simulated datasets comprising height and weight measures from 10 individuals measured yearly for 10 years (upper row), 20 individuals measured five times at two‐year intervals (middle row), and 30 individuals measured yearly for 25 years (bottom row). Plot interpretation is analogous to Appendix Figure A.2.
**Figure A.13**. Estimates of mean parameter values by age. Blue lines are means and shaded areas are 90% HPDI for posterior distributions of weighted sums of population‐mean values of model parameters *q* (allometry), *K* (catabolism), and *H* (anabolism) across the five component growth processes of the model (without incorporating age uncertainty) estimated for five independently‐simulated datasets comprising height and weight measures from 20 individuals measured five times at two‐year intervals (left column) and 30 individuals measured yearly for 25 years (right column). Plot interpretation is analogous to Figure 5 in the main text.

## Data Availability

Scripts in R and Stan to reproduce all analyses in the main text and appendices are available on Github at https://github.com/jabunce/bunce‐revilla‐fernandez‐2026‐age_uncert
