# Supplementary material for: Estimating Mean Growth Trajectories When Measurements Are Sparse and Age Is Uncertain
Source: Am J Biol Anthropol. 2026 Sep 22;191(1):e70358. doi: 10.1002/ajpa.70358 (PMC13595483; doi:10.1002/ajpa.70358)
Supplement: Supplementary file 1 [file AJPA-191-e70358-s001.pdf]

APPENDICES:

Estimating mean growth trajectories when measurements are sparse and age is uncertain

John A. Bunce*[1,2], Caissa Revilla-Minaya[1,2], & Catalina I. Fernández[2,3]

[1]Division of Anthropology, American Museum of Natural History, New York, NY USA

[2]Department of Human Behavior, Ecology, and Culture, Max Planck Institute for Evolutionary Anthropology, Leipzig, Germany

[3]Department of Anthropology, Florida Atlantic University, Boca Raton, FL USA

*Email: jbunce@amnh.org

Appendix A. Base statistical model 2
A.1. Individual-specific height uncertainty 8
Appendix B. Modeling age uncertainty 9
B.1. Individual-specific age uncertainty 11
Appendix C. Fitting the model to height alone 21
Appendix D. Fitting the model to longitudinal data 22
References 32

Appendix A. Base statistical model

Using a strategy similar to Cole (2016) when the data of interest are sparse, we fit the model to a combined dataset comprising temporally-dense longitudinal height and weight measures from 70 girls from California (Tuddenham and Snyder 1954), as well as the simulated sparse measures of interest from a population whose mean growth matches that of Matsigenka girls. For convenience, we refer to the U.S. and simulated data as coming from two different ethnic groups.

Following Bunce et al. (2025), we fit the following model of the height and weight of person j at time t to the combined dataset, with ethnic group-level (g) and person-level (p) offsets to mean parameter values ($\hat{q}$'s, $\hat{K}$'s, R's, and $\hat{i}$'s). We allow both sets of offsets to covary within and across growth phases. Person j's observed height (h_{jt}) and weight (m_{jt}) at time t are modeled as:

$$\ln(h_{jt}) \sim \text{Normal}\left[\ln(\eta_{jt}), \sigma_\eta\right] \tag{A.1}$$

$$\ln(m_{jt}) \sim \text{Normal}\left[\ln(\mu_{jt}), \sigma_\mu\right] \tag{A.2}$$

$$\eta_{jt} = 0.012 + \sum_{x=1}^{5} [t \geq i_{xj}] \left\{ \frac{2H_{xj}}{K_{xj}} \left[1 - \exp\left(\frac{K_{xj} q_{xj}}{1 + 2q_{xj}} (i_{xj} - t) \right) \right] \right\}^{\frac{1}{q_{xj}}} \tag{A.3}$$

$$\mu_{jt} = 1.02 \cdot 10^{-6} + \sum_{x=1}^{5} [t \geq i_{xj}]\, \pi \left\{ \frac{2H_{xj}}{K_{xj}} \left[1 - \exp\left(\frac{K_{xj} q_{xj}}{1 + 2q_{xj}} (i_{xj} - t) \right) \right] \right\}^{\frac{1}{q_{xj}}+2} \tag{A.4}$$

$$q_{xj} = \exp\left(\hat{q}_x + g_{q_{x,\text{ETH}[j]}} + p_{q_{xj}} \right) \tag{A.5}$$

$$K_{xj} = \exp\left(\hat{K}_x + g_{K_{x,\text{ETH}[j]}} + p_{K_{xj}} \right) \tag{A.6}$$

$$H_{xj} = \frac{K_{xj}}{2} + R_x + g_{R_{x,\text{ETH}[j]}} + p_{R_{xj}} \tag{A.7}$$

$$i_{1j} = 0, i_{x>1,j} = \exp\left(\hat{i}_x + g_{i_{x,\text{ETH}[j]}} + p_{i_{xj}} \right) \tag{A.8}$$

$$\begin{bmatrix} p_{q_1} \\ p_{q_2} \\ p_{q_3} \\ p_{q_4} \\ p_{q_5} \\ p_{K_1} \\ p_{K_2} \\ p_{K_3} \\ p_{K_4} \\ p_{K_5} \\ p_{R_1} \\ p_{R_2} \\ p_{R_3} \\ p_{R_4} \\ p_{R_5} \\ p_{i_2} \\ p_{i_3} \\ p_{i_4} \\ p_{i_5} \end{bmatrix}_j \sim \text{MVNormal}\left(\begin{bmatrix} 0 \\ 0 \\ 0 \\ 0 \\ 0 \\ 0 \\ 0 \\ 0 \\ 0 \\ 0 \\ 0 \\ 0 \\ 0 \\ 0 \\ 0 \\ 0 \\ 0 \\ 0 \\ 0 \end{bmatrix}, \mathbf{S}_{\text{ETH}[j]} \right) \tag{A.9}$$

$$\begin{bmatrix} g_{q_1} \\ g_{q_2} \\ g_{q_3} \\ g_{q_4} \\ g_{q_5} \\ g_{K_1} \\ g_{K_2} \\ g_{K_3} \\ g_{K_4} \\ g_{K_5} \\ g_{R_1} \\ g_{R_2} \\ g_{R_3} \\ g_{R_4} \\ g_{R_5} \\ g_{i_2} \\ g_{i_3} \\ g_{i_4} \\ g_{i_5} \end{bmatrix}_{\text{ETH}} \sim \text{MVNormal}\left(\begin{bmatrix} 0 \\ 0 \\ 0 \\ 0 \\ 0 \\ 0 \\ 0 \\ 0 \\ 0 \\ 0 \\ 0 \\ 0 \\ 0 \\ 0 \\ 0 \\ 0 \\ 0 \\ 0 \\ 0 \end{bmatrix}, \mathbf{T} \right) \tag{A.10}$$

$$\mathbf{S}_{\mathrm{ETH}[j]} = \begin{bmatrix} \sigma_{p_{q_1}} & 0 & . & . & . & 0 \\ 0 & . & & & & . \\ . & & . & & & . \\ . & & & . & & . \\ . & & & & . & 0 \\ 0 & . & . & . & 0 & \sigma_{p_{i_5}} \end{bmatrix}_{\mathrm{ETH}[j]} \qquad \mathbf{R}_{\mathrm{ETH}[j]} \begin{bmatrix} \sigma_{p_{q_1}} & 0 & . & . & . & 0 \\ 0 & . & & & & . \\ . & & . & & & . \\ . & & & . & & . \\ . & & & & . & 0 \\ 0 & . & . & . & 0 & \sigma_{p_{i_5}} \end{bmatrix}_{\mathrm{ETH}[j]} \tag{A.11}$$

$$\mathbf{T} = \begin{bmatrix} \sigma_{g_{q_1}} & 0 & . & . & . & 0 \\ 0 & . & & & & . \\ . & & . & & & . \\ . & & & . & & . \\ . & & & & . & 0 \\ 0 & . & . & . & 0 & \sigma_{g_{i_5}} \end{bmatrix} \mathbf{U} \begin{bmatrix} \sigma_{g_{q_1}} & 0 & . & . & . & 0 \\ 0 & . & & & & . \\ . & & . & & & . \\ . & & & . & & . \\ . & & & & . & 0 \\ 0 & . & . & . & 0 & \sigma_{g_{i_5}} \end{bmatrix} \tag{A.12}$$

$$\sigma_\eta \sim \text{Exponential}(1/0.002) \in (0, 0.005] \tag{A.13}$$
$$\sigma_\mu \sim \text{Exponential}(1/0.002) \in (0, \infty) \tag{A.14}$$

$$(\hat{q_1}, \hat{q_2}, \hat{q_3}, \hat{q_4}, \hat{q_5}) = (-2.47, -1.85, -1.19, -3.02, -2.10) \tag{A.15}$$
$$\left(\hat{K_1}, \hat{K_2}, \hat{K_3}, \hat{K_4}, \hat{K_5}\right) = (4.40, 2.51, 0.45, 2.38, 2.18) \tag{A.16}$$
$$(R_1, R_2, R_3, R_4, R_5) = (16.66, 4.29, 1.84, 0.66, 1.80) \tag{A.17}$$
$$\left(\hat{i_2}, \hat{i_3}, \hat{i_4}, \hat{i_5}\right) = (-2.11, -0.30, 0.93, 2.26) \tag{A.18}$$

$$\sigma_{p_{q_x},\mathrm{ETH}} \sim \text{Normal}(0.003, 0.003) \in (0, \infty) \tag{A.19}$$
$$\sigma_{p_{K_x},\mathrm{ETH}} \sim \text{Normal}(0.003, 0.003) \in (0, \infty) \tag{A.20}$$
$$\sigma_{p_{R_x},\mathrm{ETH}} \sim \text{Normal}(0.003, 0.003) \in (0, \infty) \tag{A.21}$$
$$\sigma_{p_{i_{x>1}},\mathrm{ETH}} \sim \text{Normal}(0.003, 0.003) \in (0, \infty) \tag{A.22}$$
$$\mathbf{R}_{\mathrm{ETH}} \sim \text{LKJcorr}(4) \tag{A.23}$$

$$\sigma_{g_{q_x}} \sim \text{Normal}(0.003, 0.001) \in (0, \infty) \tag{A.24}$$
$$\sigma_{g_{K_x}} \sim \text{Normal}(0.003, 0.001) \in (0, \infty) \tag{A.25}$$
$$\sigma_{g_{R_x}} \sim \text{Normal}(0.003, 0.001) \in (0, \infty) \tag{A.26}$$
$$\sigma_{g_{i_{x>1}}} \sim \text{Normal}(0.003, 0.001) \in (0, \infty) \tag{A.27}$$
$$\mathbf{U} \sim \text{LKJcorr}(4) \tag{A.28}$$

where the subscript ETH[j] refers to the ethnicity (U.S. or Matsigenka)

of individual j. In practice, the covariance matrices $\mathbf{S}$ and $\mathbf{T}$ are fit using Cholesky decomposition (McElreath 2020). As explained in Bunce et al. (2025, Appendix D.1.3), fitting the model to height and weight data simultaneously requires allowing measurement error for weight (σ_μ) to be greater than that for height (σ_η). Thus, the bounds in equation A.14 are wider than in equation A.13. An upper bound of 0.005 on σ_η corresponds, on the scale of the data, to measurement error such that approximately 95% of measurements fall within 2*stdev = 1 cm of the actual height when height = 100 cm. Priors on these measurement error terms are explained in Bunce et al. (2025, Appendix D.2). Priors on the transformed parameters, $\hat{q}$'s, $\hat{K}$'s, R's, and $\hat{i}$'s, are derived from fitting the model to data from U.S. girls (Bunce et al. 2025).

The ethnic group-specific individual-level standard deviations (σ_p's), as well as the the group-level standard deviations (σ_g's), are sampled from Gaussian distributions truncated at zero. The means of these prior distributions are chosen simply to yield adequate model convergence and fit, as we currently have limited prior information about how much variation exists between different human populations with respect to q, K, H, and i. Priors on the correlation matrices $\mathbf{R}$ and $\mathbf{U}$ are chosen to bias against extreme correlations (McElreath 2020).

Models were fit in R (R Core Team 2022) and Stan (Stan Development Team 2022) using the cmdstanr package (Gabry and Cesnovar 2021). Convergence was indicated by $\hat{R}$ values of 1.00. This usually required four chains of 4000 samples each, half of which were warm-up. In some model estimates, an $\hat{R}$ of 1.01 for no more than one parameter, where all other parameters had an $\hat{R}$ of 1.00, was also accepted as indicative of convergence. Data and analysis scripts are available at `https://github.com/jabunce/bunce-revilla-fernandez-2026-age_uncert`.

Posterior parameter estimates for each of the five growth processes are shown in Figures A.1 and A.2, for simulated cross-sectional datasets of different sizes.

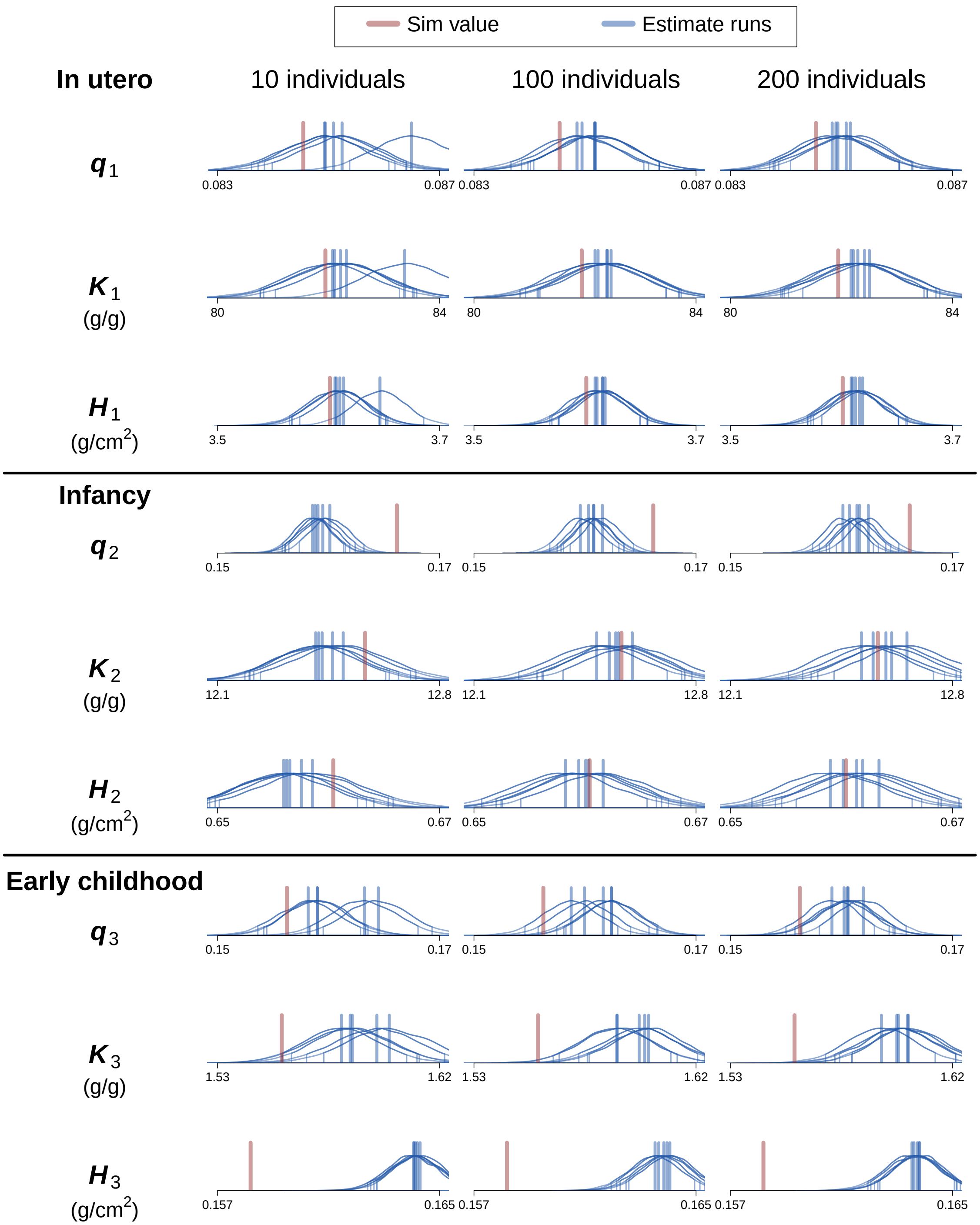

FIGURE A.1. (Continued on next page.)

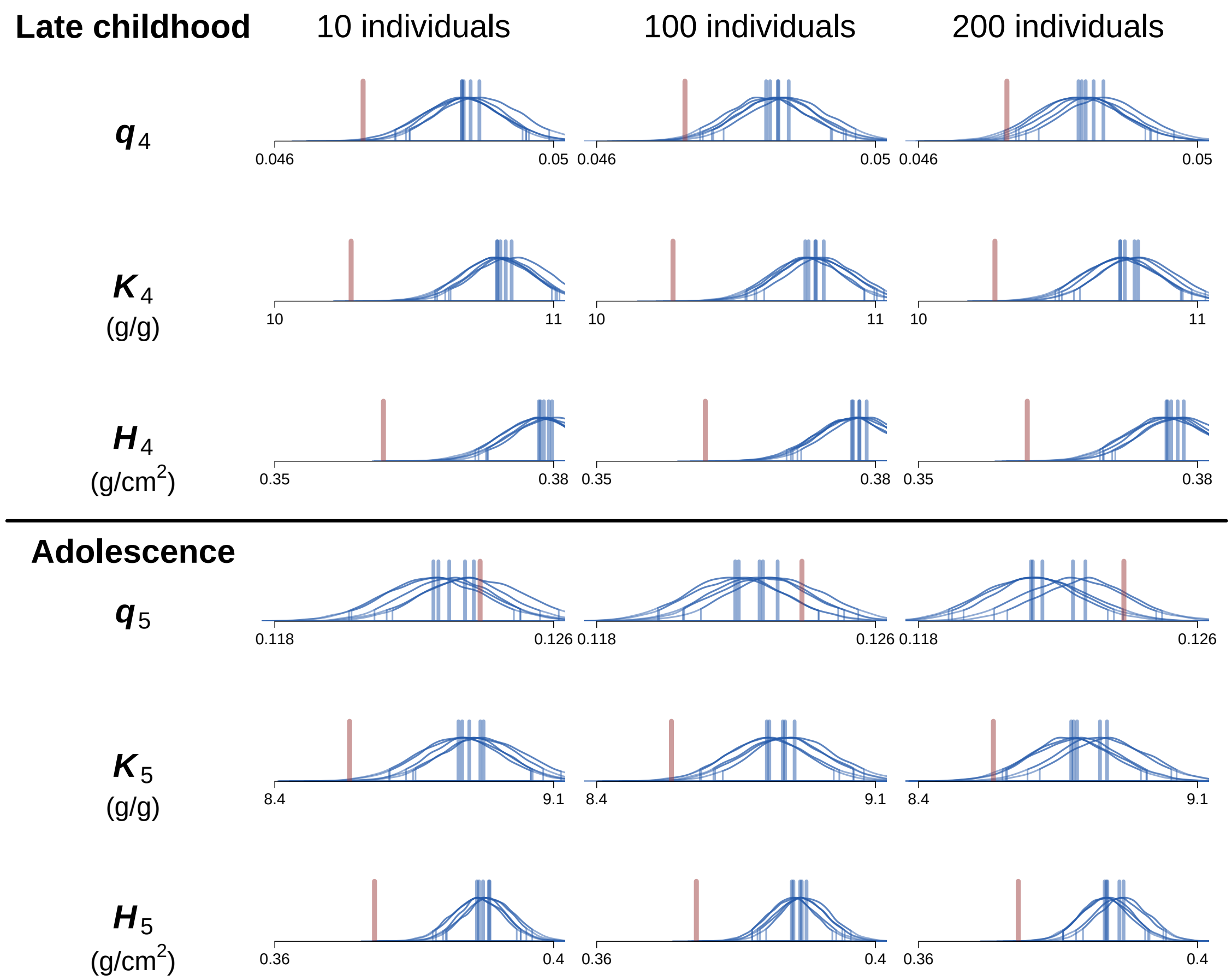

FIGURE A.1. (Continued from previous page) Posterior estimates of population-mean parameter values for each of the five growth processes in the composite growth model (Bunce et al. 2025, Appendix C), when fit to simulated cross-sectional datasets from 10 (left column), 100 (center column), and 200 (right column) individuals. Five posterior distributions (blue) come from fitting the model to five independently-simulated cross-sectional datasets for each sample size. Vertical blue lines are means of each posterior distribution, and marked distribution tails enclose 90% highest posterior density intervals (HPDIs). HPDIs that overlap the actual population-mean value of the simulated datasets (red vertical lines) indicate accurate, though not necessarily precise, posterior estimates.

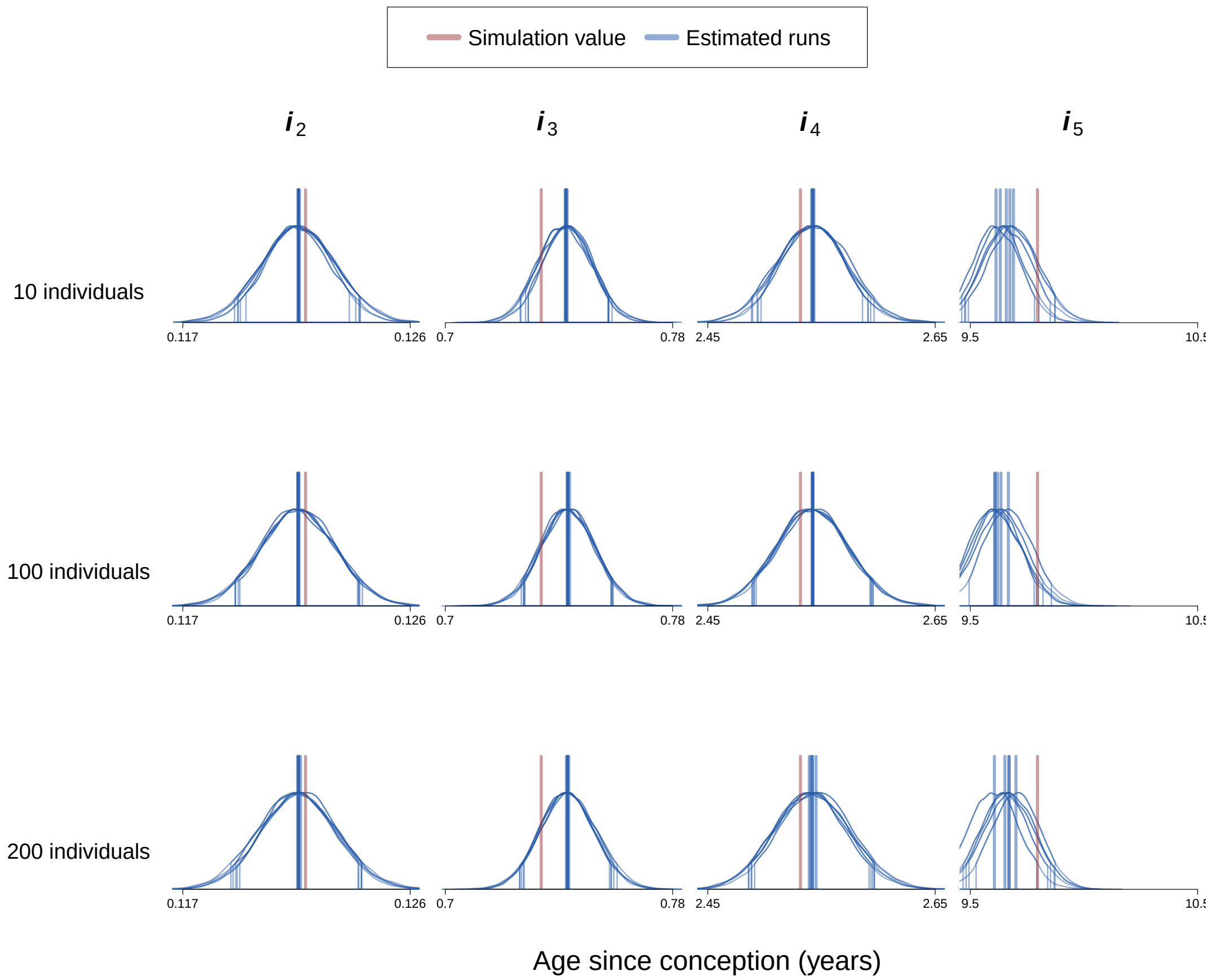

FIGURE A.2. Posterior estimates of population-mean values of initiation parameters, i, for each of the five growth growth processes (columns), when fit to simulated cross-sectional datasets from 10 (top row), 100 (center row), and 200 (bottom row) individuals. Five posterior distributions (blue) come from fitting the model to five independently-simulated cross-sectional datasets for each sample size. Vertical blue lines are means of each posterior distribution, and marked distribution tails enclose 90% HPDIs. HPDIs that overlap the actual population-mean value of the simulated datasets (red vertical lines) indicate accurate, though not necessarily precise, posterior estimates.

A.1. **Individual-specific height uncertainty.** It may be the case that not all individuals are subject to the same uncertainty parameters

for height and weight. For instance, it may be known that measurement error in height differs between two individuals who are recorded to have the same height. Individual-specific uncertainty can be incorporated into the statistical model by replacing σ_η in equation A.1 with the vector $\sigma_{\eta j}$, where each element is a standard deviation unique to individual j.

For instance, assume a cross-sectional dataset (e.g., in a bioarchaeological context) where uncertainty due to measurement error is unique to each individual's single height measurement, and is random with respect to true height. This could be represented as a Gaussian distribution whose mean equals j's (unknown) true height (η_{jt}), and whose standard deviation is such that the limits of a j-specific range in measurement error (e.g., ±5cm) correspond to two standard deviations away from this mean. To find the value of $\sigma_{\eta j}$ on the log scale that results in such a distribution, the following formula can be used:

$$\sigma_{\eta j} = \sqrt{\ln\left(\frac{1+\sqrt{1+\frac{4(\frac{1}{2}s)^2}{h^2}}}{2}\right)} \tag{A.29}$$

where h is the single measured height of individual j, and s is the j-specific error limit (e.g., 5cm) corresponding to two standard deviations away from the mean. See also Appendix B.1, below.

Appendix B. Modeling age uncertainty

To incorporate age uncertainty into the model, instead of treating total age t as data, we instead treat it as a parameter to be estimated. For all individuals j such that ETH$[j] = 2$, where 2 is the index representing the simulated (Matsigenka-like) data, total age since conception, t, is modeled as:

$$t = y_m - y_c \tag{A.30}$$

$$y_c \sim \text{Normal}\left(y_o, \sigma_\epsilon\right) \tag{A.31}$$

$$\sigma_\epsilon = a_o \cdot \epsilon \tag{A.32}$$

where y_m is the year of measurement (which is data), y_c is the true year of conception, which is sampled from a Normal distribution whose

mean, y_o is the potentially-inaccurate recorded year of conception (recorded year of birth - 0.75), which is data, and whose standard deviation is the product of the recorded age at measurement, a_o (data), and the uncertainty factor ϵ, which is set to 0.1 for all analyses. Note that this strategy is analogous to how age uncertainty is incorporated into the data simulations (main text Material and Methods: Simulation of Age Uncertainty).

Figure A.3 illustrates how model posterior mean estimates of total age t (red circles) compare to observed ages (blue circles) and the actual ages (black points) of the simulated dataset. Note that error in the observed data (distance between blue circles and black points) is simulated to increase with age, and that this error is randomly distributed as older or younger than the actual age (blue circles are randomly either left or right of the black points), analogous to Figure 2 in the main text. In contrast, note that the means of the posterior age estimates (red circles) are always shifted (from the blue circles, which are the data) closer to the population mean growth trajectory, and that the magnitude of such shifts increases with age. This is a manifestation of the conservative assumption that, if we believe a child's observed (or inferred) growth trajectory is inaccurate due to error in measurement of the child's age, then, in the absence of any additional information, our best guess about the child's true growth trajectory should be that it more closely resembles the population-mean growth trajectory. Additionally, the older the child at the time of first measurement, the more error we should suspect in the measurement of her age (main text Material and Methods: Simulation of Age Uncertainty).

Figure A.3 shows trajectories, Figure A.4 shows trajectory characteristics, and Figures A.6 and A.7 show parameters estimated from the model with age uncertainty fit to cross-sectional height and weight data from 100 individuals ("100 individuals, estimated age"). Comparing these figures with Figures 3 and 4 in the main text and Appendix Figures A.1 and A.2 reveals no substantial increase in the accuracy of estimates of parameters and the population mean growth trajectory compared to a model that ignores age uncertainty. This is a result of the assumption, during both data simulation and model design, that uncertainty in age is random with respect to direction. In other words, it is just as likely that a child's age has been underestimated as overestimated, and the magnitude of any such under- or over-estimation is comparable (for a given age). This is a strong assumption, and ensures that age uncertainty has no effect, on average, on estimates of the population mean growth trajectory. For actual datasets, fieldworkers may have more information about the data-generating process (e.g., that

results in errors during age recording) permitting more sophisticated modeling of age uncertainty. Equations A.30 - A.32 can be modified to reflect such knowledge, and may have a profound effect on the accuracy of the estimated population mean growth trajectory (see also McElreath (2020), pgs 491-499).

B.1. **Individual-specific age uncertainty.** This statistical model requires you to choose your best guess for an individual's age. For instance, given an age range of 8-11 years for individual j, your best guess about j's actual age might be the midpoint (9.5 years). This would be the data point for j's "observed" age. The model will give you its best estimate of j's actual age once it knows your uncertainty, in this case ± 1.5 years on either side of the observed age. You can then choose a standard deviation specific to j (i.e., $\sigma_{\epsilon j}$ in equation A.31) such that twice the standard deviation is equal to 1.5. In this case $\sigma_{\epsilon j}$ $= 1.5/2 = 0.75$. This tells the model that you expect the true value of j's age to have a 95.4% chance (i.e., two standard deviations) of falling within a range of 1.5 years on either side of 9.5 years. Note that, unlike for height and weight (Appendix A.1, above), it is not necessary to transform the standard deviation in age onto the log scale.

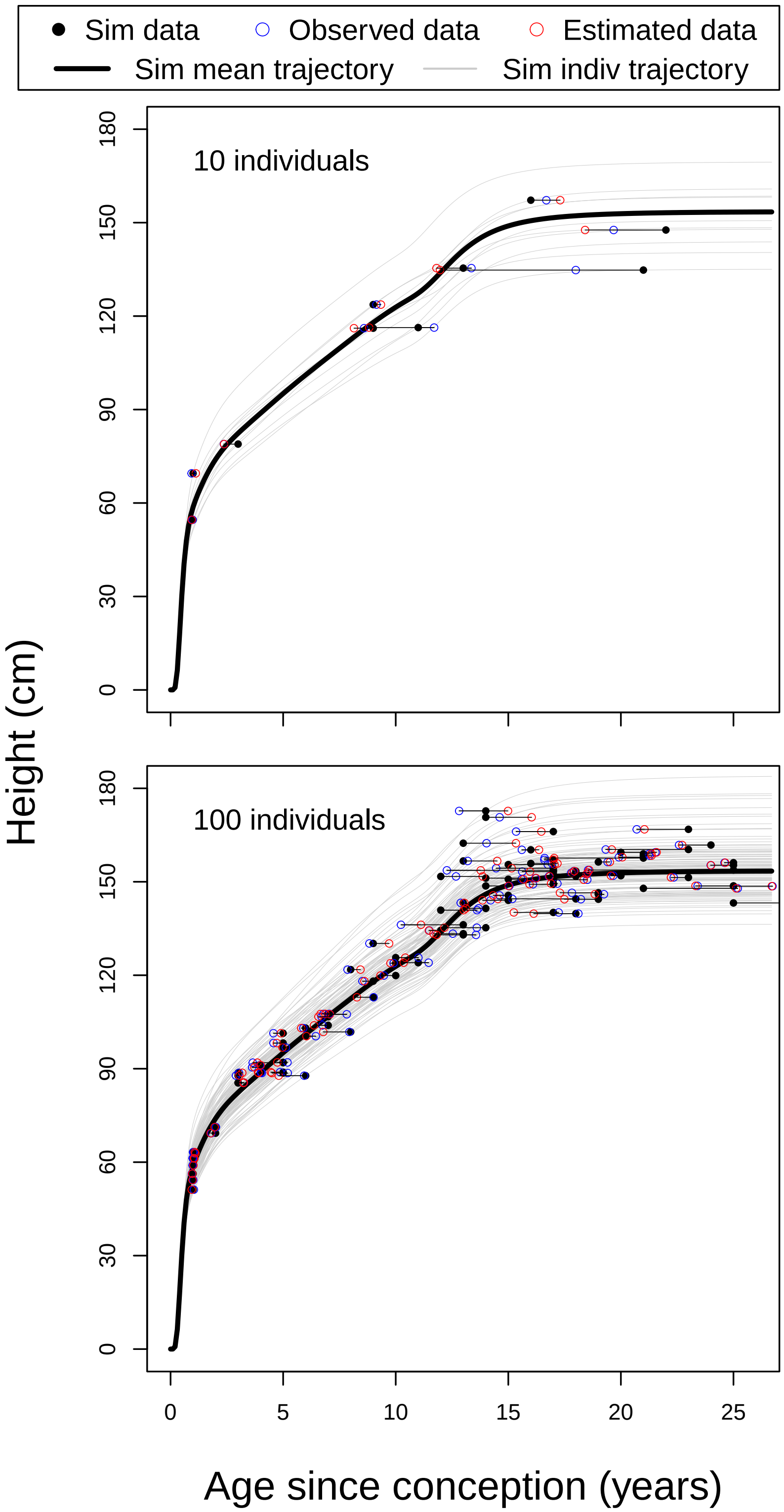

FIGURE A.3. (Caption next page.)

FIGURE A.3. Simulated cross-sectional height data for 100 individuals (black points), where thick black lines are the true population-mean growth trajectories and grey lines are individual trajectories. Horizontal lines connect each point to the potentially-inaccurate "observed" data for that individual (blue circles), which incorporate uncertainty in the age recorded for each child during height measurement. Horizontal lines also connect each observed data point to the posterior mean location of each data point estimated by the model incorporating age uncertainty (red circles). Note that model estimates of data points tend to be shifted toward the population mean trajectory relative to the observed data. See also Figure 1 in the main text.

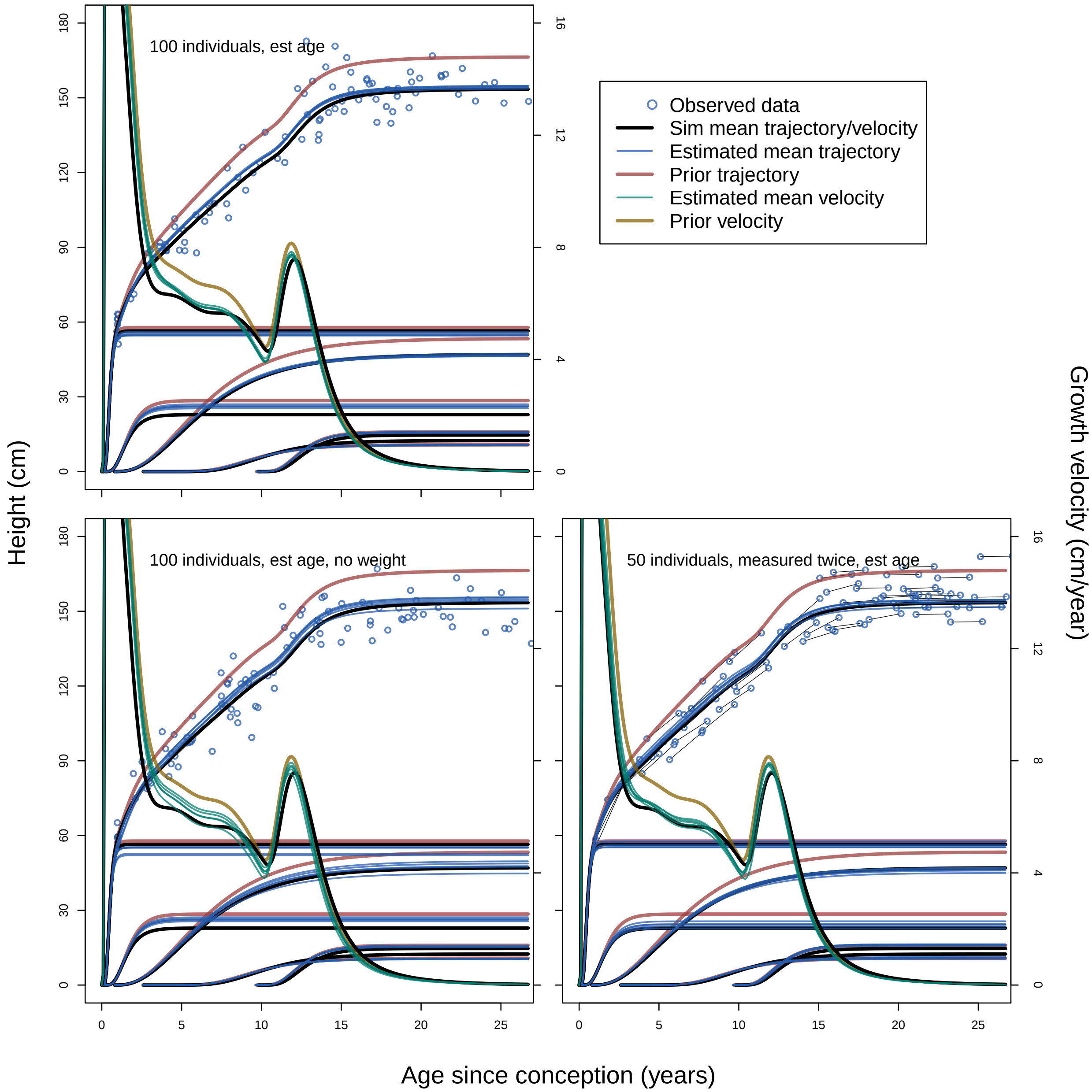

FIGURE A.4. (Caption next page.)

Figure A.4. Mean posterior cumulative population-mean height growth trajectories and component functions (blue lines) estimated using a model incorporating age uncertainty and applied to five independently-simulated datasets for each of three measurement strategies: single height and weight measures from 100 individuals (upper left), single height (but not weight) measures from 100 individuals (lower left), and height and weight measures from 50 individuals, each measured twice at an interval of two years (lowe right). Data points (observed rather than true: Figure 2) in each plot come from one of the five simulations for a dataset of a given type. Plot interpretation is analogous to Figure 3 in the main text.

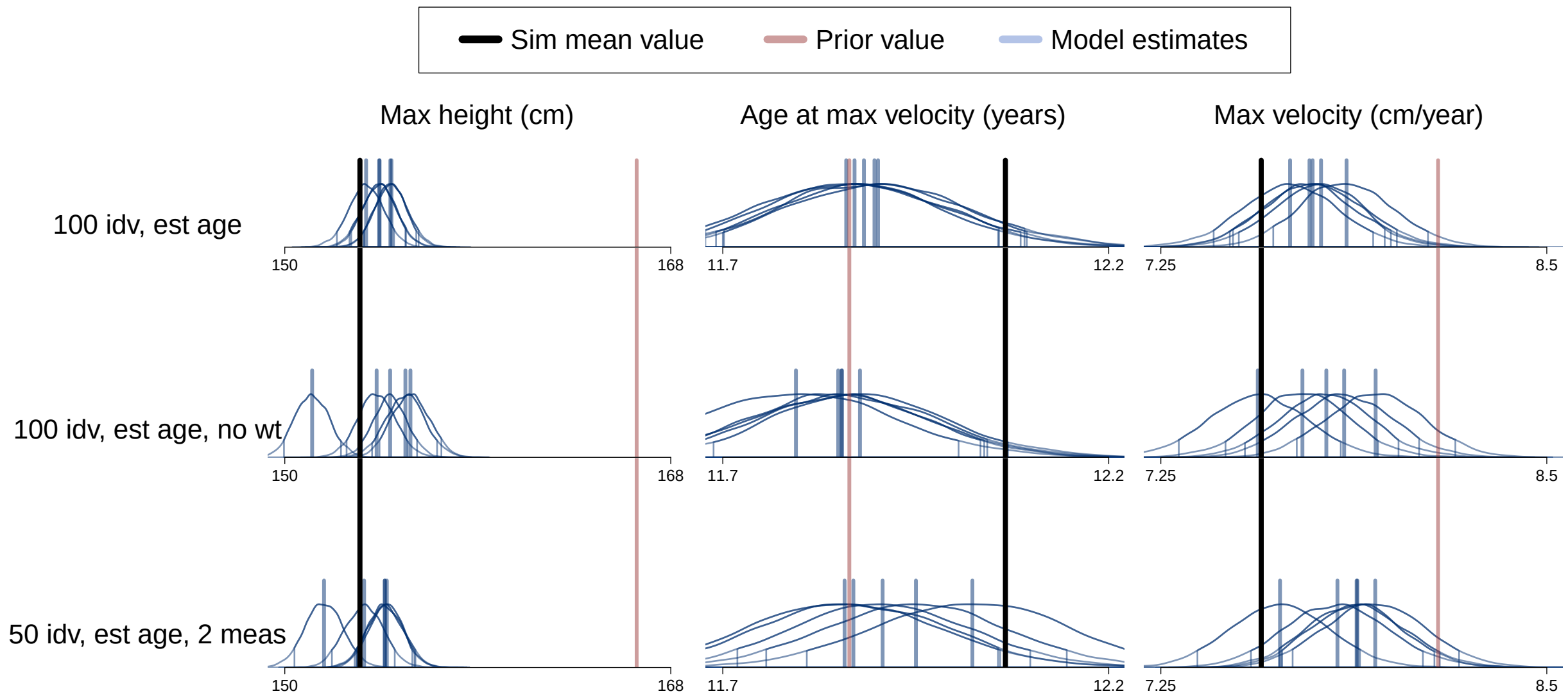

Figure A.5. Three characteristics of the population-mean height growth trajectories illustrated in Appendix Figure A.4: maximum achieved height (left column), age since conception at which the maximum growth velocity during puberty is observed (center column), and maximum growth velocity during puberty (right column). Five posterior distributions (blue) come from fitting the composite model incorporating age uncertainty to five independently-simulated datasets comprising single height and weight measures from 100 individuals (top row), single height (but not weight) measures from 100 individuals (center row), and height and weight measures from 50 individuals, each measured twice at an interval of two years (bottom row). Plot interpretation is analogous to Figure 4 in the main text.

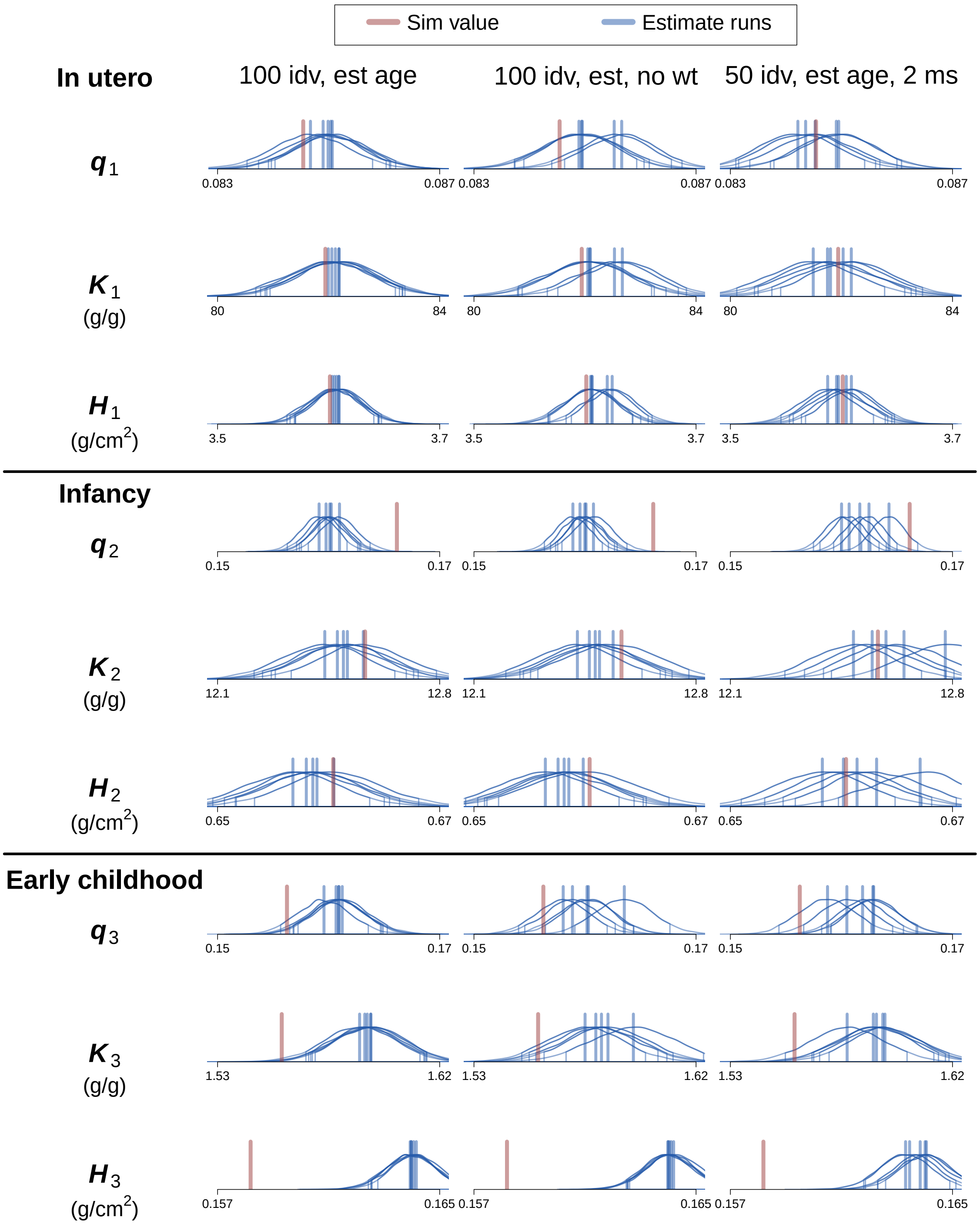

FIGURE A.6. (Continued on next page.)

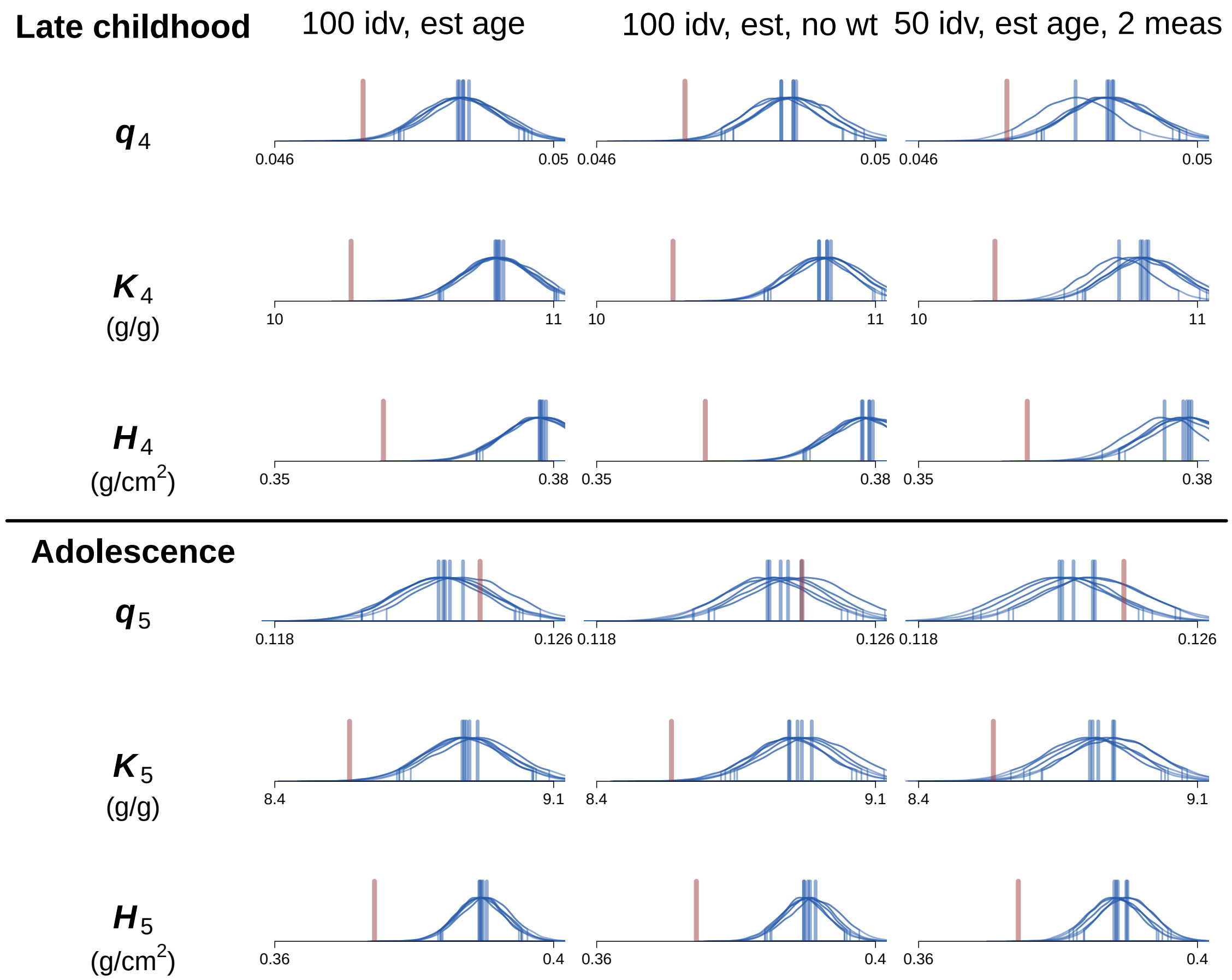

Figure A.6. (Continued from previous page) Posterior estimates of population-mean parameter values for each of the five growth processes in the composite model incorporating age uncertainty, when fit to simulated datasets comprising single height and weight measures from 100 individuals (left column), single height (but not weight) measures from 100 individuals (center column), and height and weight measures from 50 individuals, each measured twice at an interval of two years (right column). Plot interpretation is analogous to Appendix Figure A.1.

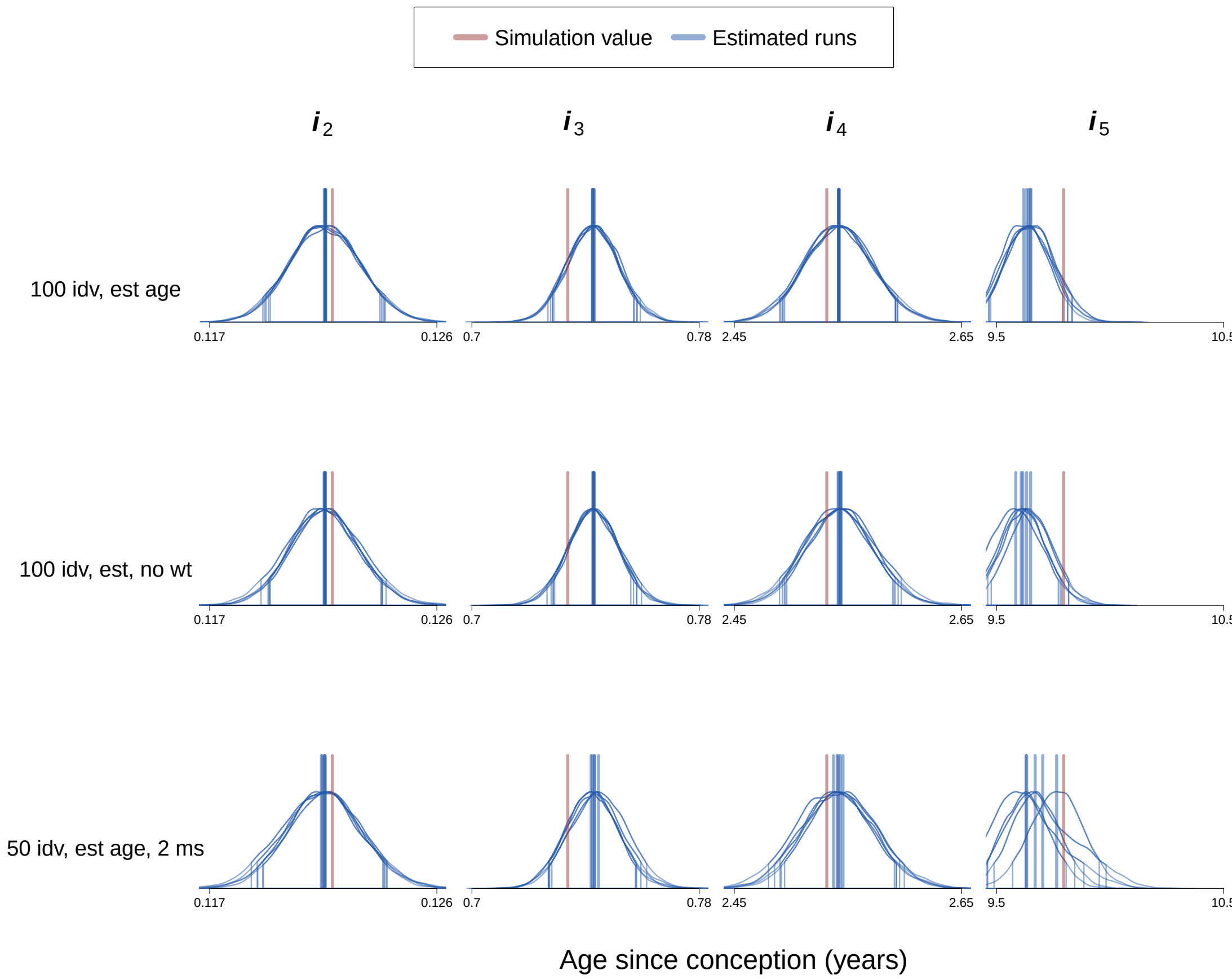

FIGURE A.7. Posterior estimates of population-mean values of initiation parameters, i, for each of the five growth growth processes (columns), when fit to simulated datasets comprising single height and weight measures from 100 individuals (upper row), single height (but not weight) measures from 100 individuals (middle row), and height and weight measures from 50 individuals, each measured twice at an interval of two years (bottom row). Plot interpretation is analogous to Appendix Figure A.2.

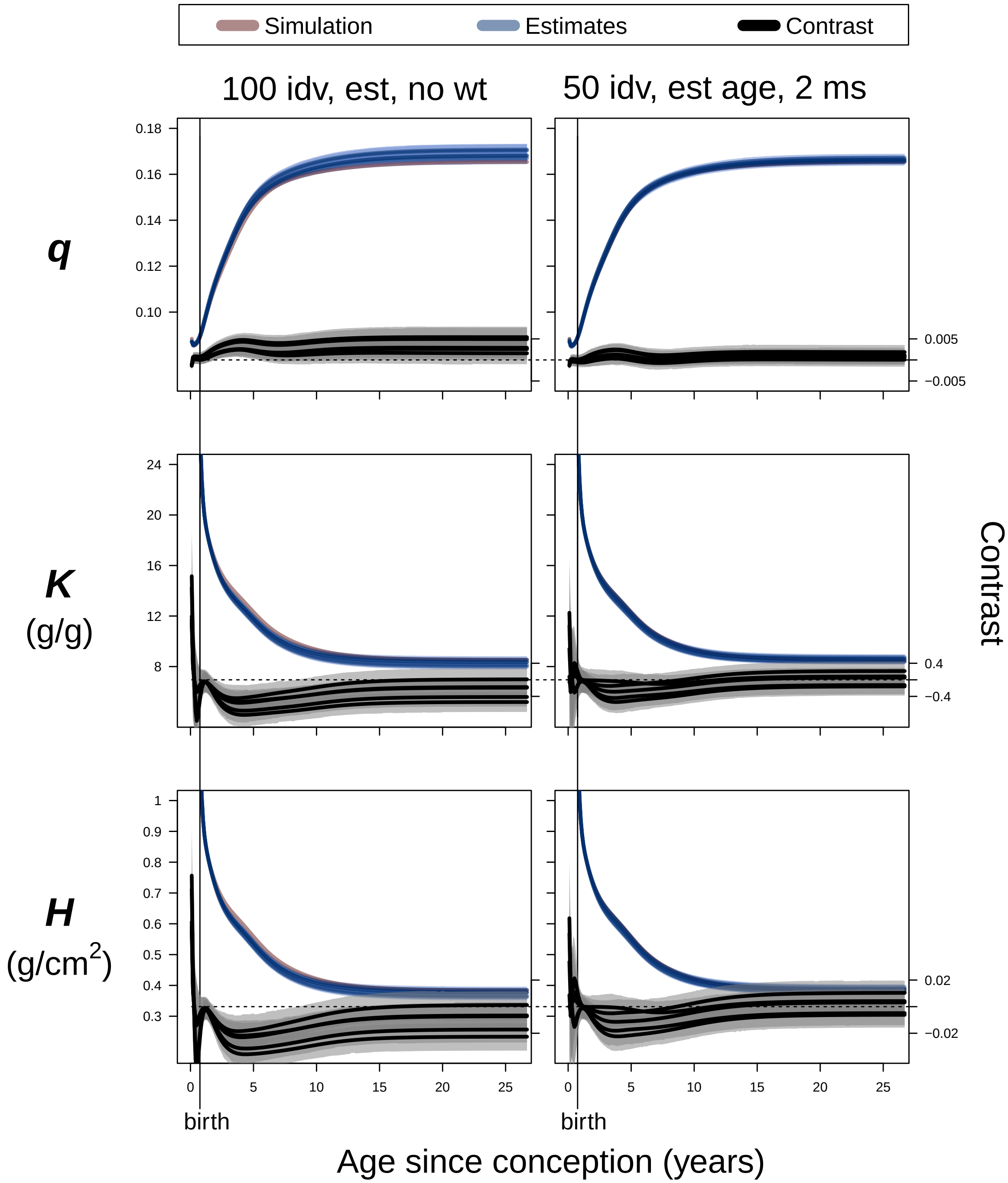

FIGURE A.8. (Caption next page.)

FIGURE A.8. Estimates of mean parameter values by age. Blue lines are means and shaded areas are 90% HPDI for posterior distributions of weighted sums of population-mean values of model parameters q (allometry), K (catabolism), and H (anabolism) across the five component growth processes of the model incorporating age uncertainty estimated for five independently-simulated datasets comprising single height (but not weight) measures from 100 individuals (left column), and height and weight measures from 50 individuals, each measured twice at an interval of two years (right column). Plot interpretation is analogous to Figure 5 in the main text.

APPENDIX C. FITTING THE MODEL TO HEIGHT ALONE

Bunce et al. (2025, Appendix D.1.3) explains why fitting the model to height and weight simultaneously is likely to result in more accurate estimates of the growth trajectory, and also why estimates of weight trajectories produced by this model are likely to be less accurate than height trajectories. Logistical challenges may prevent collecting both height and weight measures from children. Thus, if only one type of data can be collected, heights are preferred, and it is of interest to know how much accuracy is lost in such a situation. Here, we fit the model to a combined dataset consisting of heights from the simulated data, and heights and weights from the U.S. Following the strategy of McElreath (2020, pgs 499-512), we impute the weights of the simulated individuals from whom we only have heights.

Figure A.3 shows trajectories, Figure A.4 shows trajectory characteristics, Figures A.6 and A.7 show individual parameters, and Figure A.13 shows combined parameters estimated from the model with age uncertainty fit to cross-sectional height (not weight) data from 100 individuals ("100 individuals, estimated age, no weight"). Comparing these figures with Figures 3 and 4 and Appendix Figures A.1 and A.2 reveals no substantial decrease in the accuracy of estimates of parameters and the population mean growth trajectory compared to a model that includes weight for these 100 individuals. Note that, even in the absence of weight measures for the simulated individuals, the model is still simultaneously fit to height and weight measures of the U.S. children. This likely prevents a substantial decrease in the accuracy of model estimates compared to fitting the full dataset.

Appendix D. Fitting the model to longitudinal data

Thus far, the simulated data have been cross-sectional, i.e., the height (and weight) of each child is measured at a single point in time. This represents a context in which logistical challenges prevent longitudinal (repeated) measures of children across multiple years. Alternatively, it could represent a bio-archaeological dataset where heights, weights, and ages are measured (inferred) at death. In contexts in which the collection of longitudinal growth data is difficult but feasible, it is of interest to know how to design a sampling strategy that most productively balances data collection effort against accuracy of estimation of the population mean growth trajectory.

Here we consider four strategies, each resulting in 100 data points: 1) collect single measures from 100 children; 2) measure 50 children twice, with a two-interval between measures; 3) measure 10 children 10 times at one-year intervals; and 4) measure 20 children five times at two-year intervals. Model estimates using strategies 1 and 2 are illustrated in Appendix Figures A.4 - A.8 (where "100 individuals, estimated age" and "100 individuals, estimated age, no weight" are considered comparable). Model estimates using strategies 3 and 4 are illustrated in Appendix Figures A.9 - A.13. Note that strategies and 1 and 2 are fit with models that incorporate age uncertainty, while strategies 3 and 4 are fit with models that do not. As demonstrated in Appendix B, modeling age uncertainty in this context is not expected to substantially influence uncertainty in estimates of the population mean growth trajectory.

As can be seen in these figures, collecting 100 data points using a longitudinal, rather than cross-sectional, sampling strategy often results in more accurate estimates of the population mean trajectory, particularly for estimates of the age at which maximum growth velocity is achieved during puberty (Appendix Figures A.5 and A.10). In this regard, strategy 4, measuring 20 children five times at two-year intervals, would be the recommended strategy. However, none of the longitudinal strategies appear to provide a clear improvement in accuracy over a cross-sectional strategy with regard to estimates of maximum achieved height and the maximum achieved velocity during puberty (Appendix Figures A.5 and A.10). With regard to the accuracy of overall estimates for parameters q, H, and K, strategy 2, entailing double measures from 50 children, appears slightly more accurate, on average, than strategy 4 (Appendix Figures A.8 and A.13).

Comparing these four strategies against a dense longitudinal sampling strategy entailing yearly measures of 30 children from birth to

age 25, shows that while such a strategy does markedly improve accuracy of model estimates of the age of maximum growth velocity at puberty (Appendix Figure A.10) and overall parameter estimates (Appendix Figure A.13), it does not provide a clear advantage over most other strategies with regard to estimating maximum achieved height and growth velocity during puberty (Appendix Figure A.13) and estimates of individual model parameters (Appendix Figures A.11 and A.12).

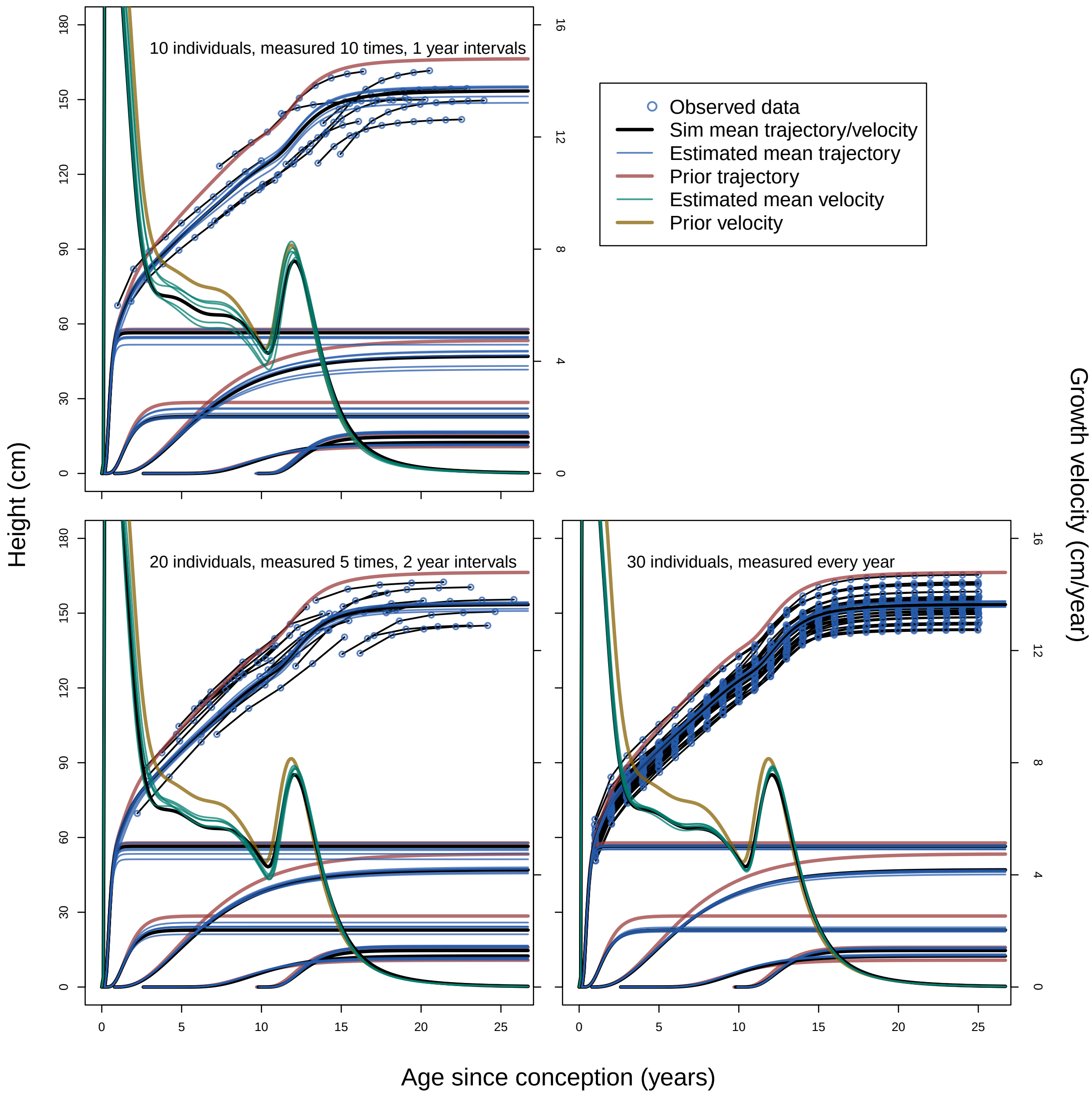

Figure A.9. (Caption next page.)

FIGURE A.9. Mean posterior cumulative population-mean height growth trajectories and component functions (blue lines) estimated using a model (without incorporating age uncertainty) applied to five independently-simulated datasets for each of three measurement strategies: height and weight measures from 10 individuals measured yearly for 10 years (upper left), 20 individuals measured five times at two-year intervals (lower left), and 30 individuals measured yearly for 25 years (lower right). Data points (observed rather than true: Figure 2) in each plot come from one of the five simulations for a dataset of a given type. Plot interpretation is analogous to Figure 3 in the main text.

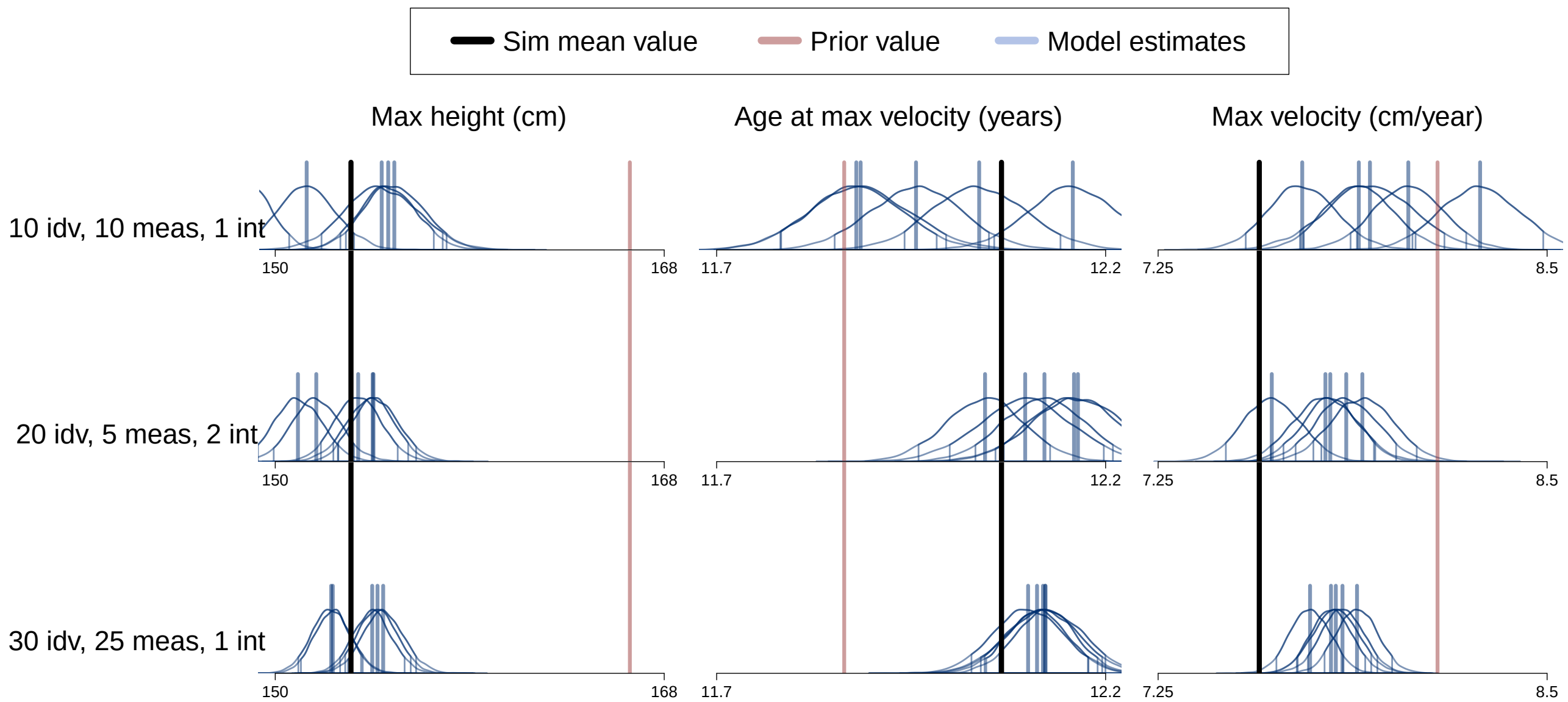

FIGURE A.10. Three characteristics of the population-mean height growth trajectories illustrated in Appendix Figure A.9: maximum achieved height (left column), age since conception at which the maximum growth velocity during puberty is observed (center column), and maximum growth velocity during puberty (right column). Five posterior distributions (blue) come from fitting the composite model (without incorporating age uncertainty) to five independently-simulated datasets comprising height and weight measures from 10 individuals measured yearly for 10 years (top row), 20 individuals measured five times at two-year intervals (middle row), and 30 individuals measured yearly for 25 years (bottom row). Plot interpretation is analogous to Figure 4 in the main text.

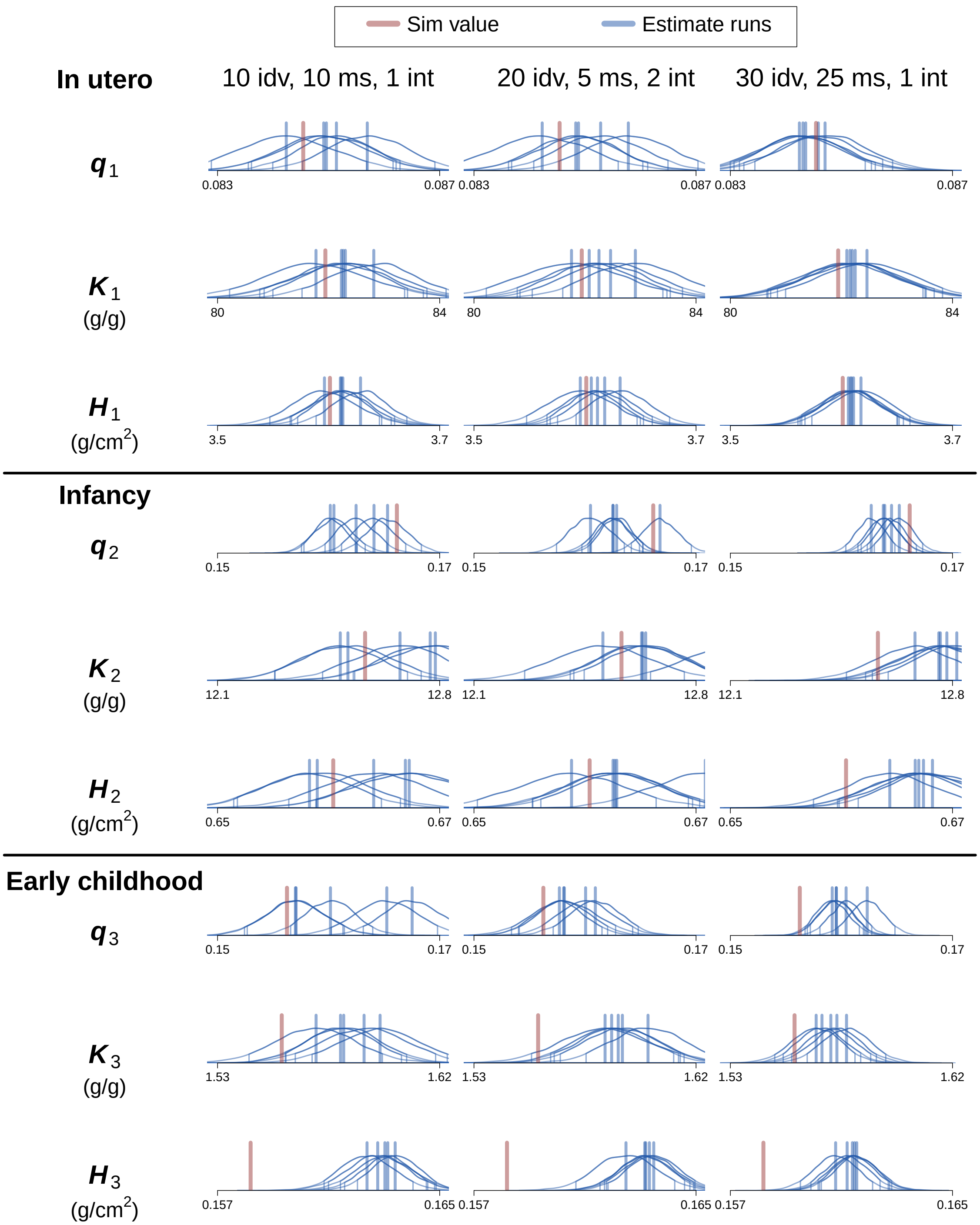

FIGURE A.11. (Continued on next page.)

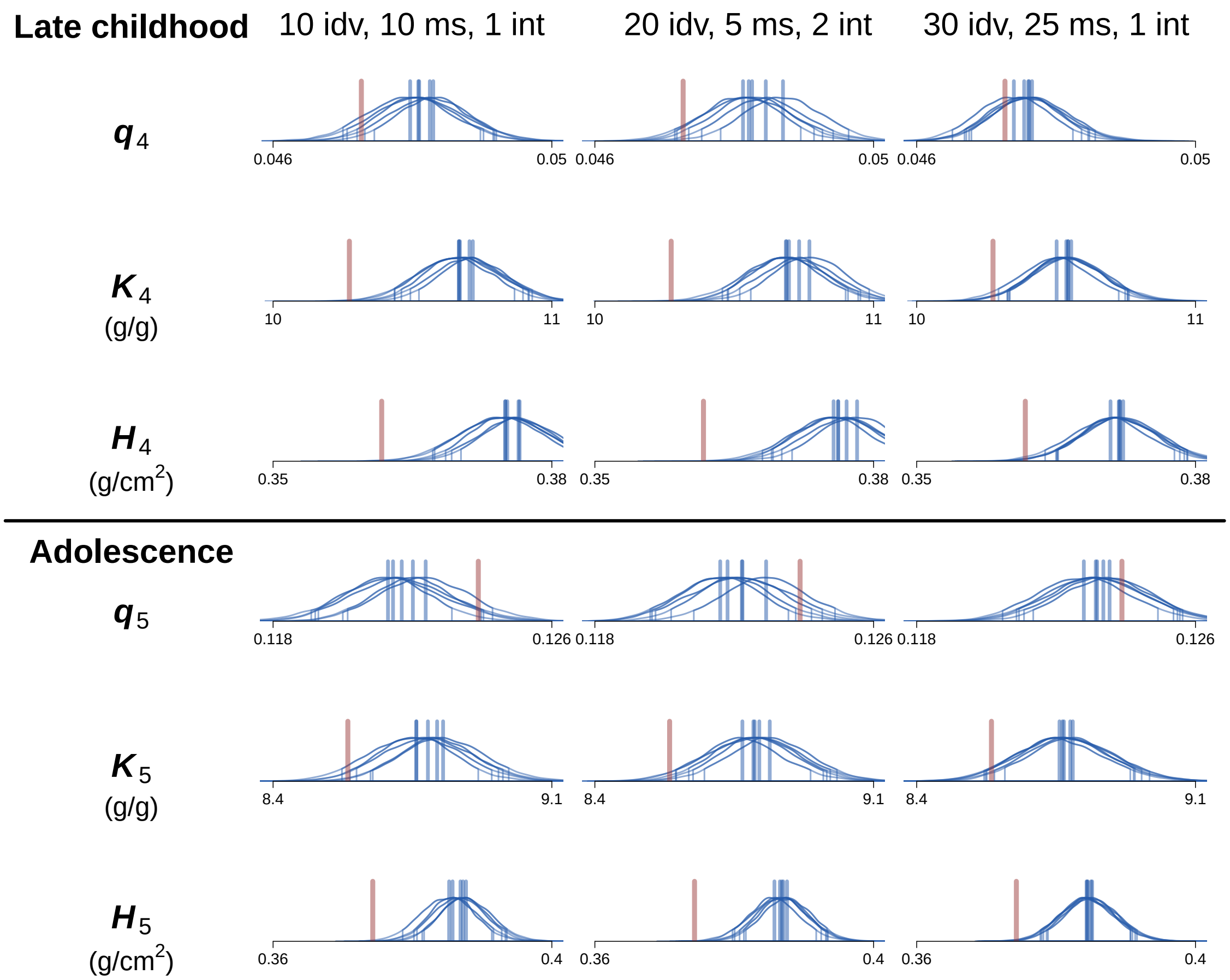

Figure A.11. (Continued from previous page) Posterior estimates of population-mean parameter values for each of the five growth processes in the composite model (without incorporating age uncertainty) when fit to simulated datasets comprising height and weight measures from 10 individuals measured yearly for 10 years (left column), 20 individuals measured five times at two-year intervals (middle column), and 30 individuals measured yearly for 25 years (right column). Plot interpretation is analogous to Appendix Figure A.1.

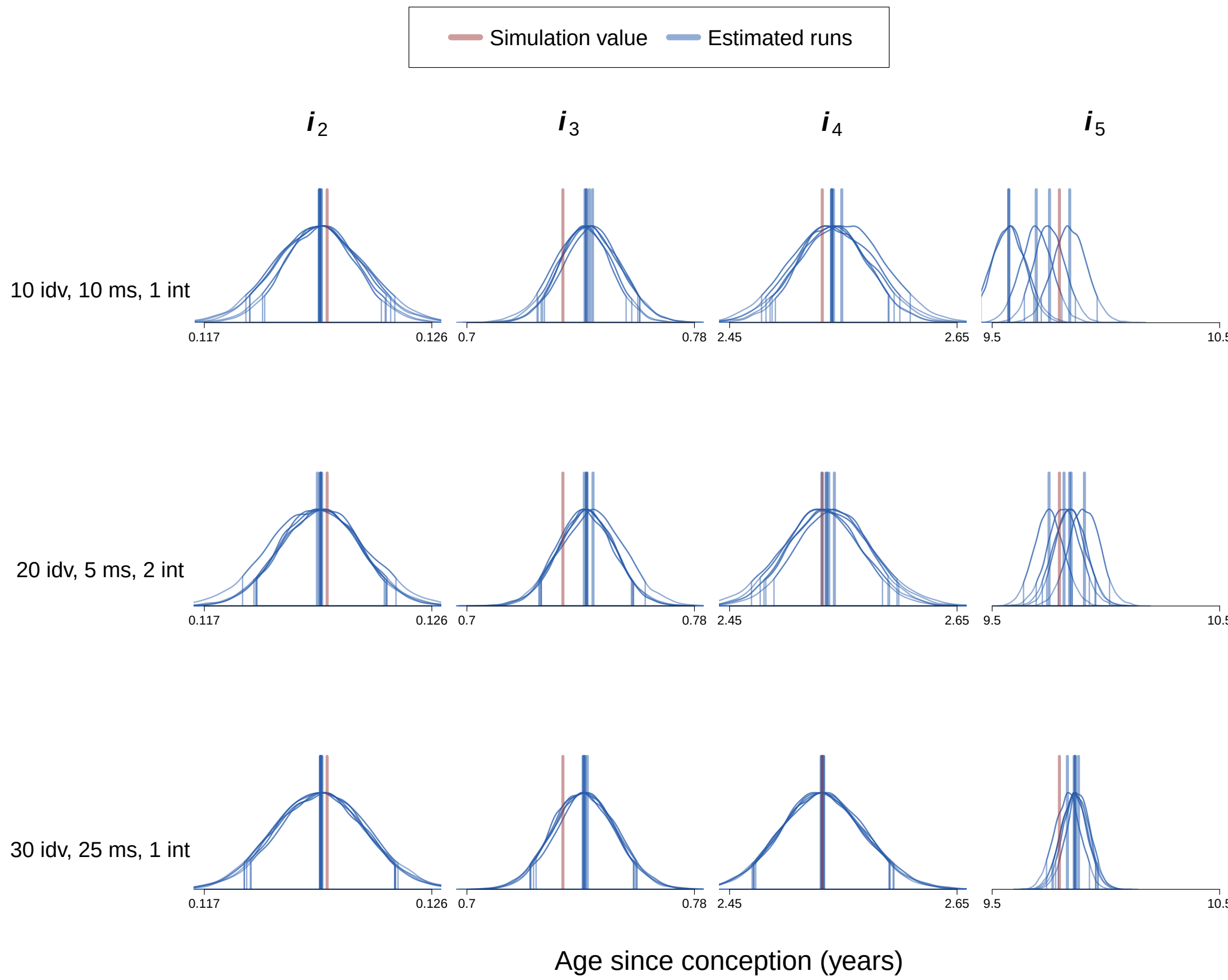

FIGURE A.12. Posterior estimates of population-mean values of initiation parameters, i, for each of the five growth growth processes (columns), when the model (without incorporating age uncertainty) is fit to simulated datasets comprising height and weight measures from 10 individuals measured yearly for 10 years (upper row), 20 individuals measured five times at two-year intervals (middle row), and 30 individuals measured yearly for 25 years (bottom row). Plot interpretation is analogous to Appendix Figure A.2.

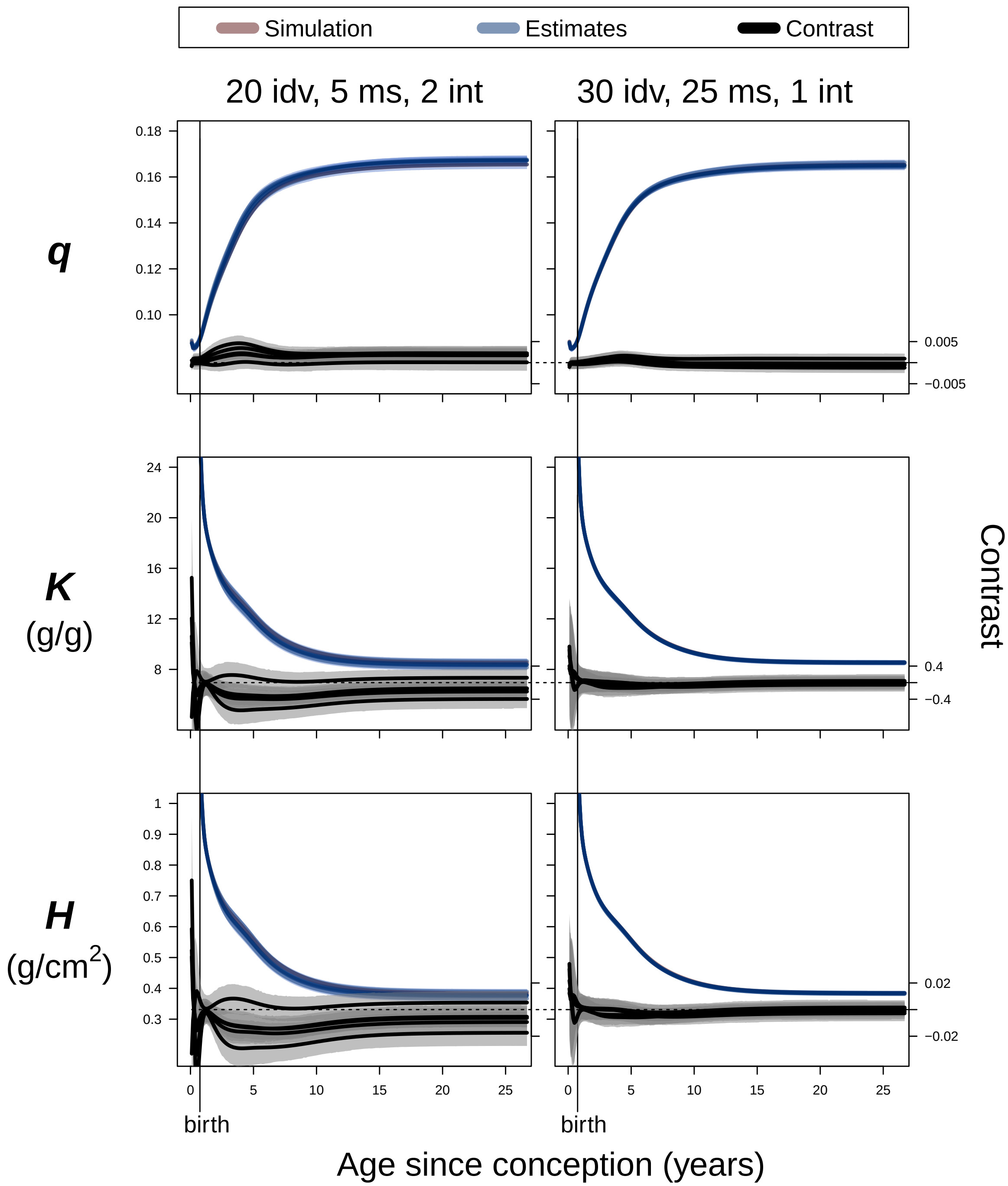

FIGURE A.13. (Caption next page.)

FIGURE A.13. Estimates of mean parameter values by age. Blue lines are means and shaded areas are 90% HPDI for posterior distributions of weighted sums of population-mean values of model parameters q (allometry), K (catabolism), and H (anabolism) across the five component growth processes of the model (without incorporating age uncertainty) estimated for five independently-simulated datasets comprising height and weight measures from 20 individuals measured five times at two-year intervals (left column) and 30 individuals measured yearly for 25 years (right column). Plot interpretation is analogous to Figure 5 in the main text.

References

Bunce, J.A., Fernández, C.I., Revilla-Minaya, C., 2025. A causal model of human growth and its estimation using temporally sparse data. Royal Society Open Science 12, 250084.

Cole, T., Kuh, D., Johnson, W., Ward, K., Howe, L., Adams, J., Hardy, R., Ong, K., 2016. Using super-imposition by translation and rotation (SITAR) to relate pubertal growth to bone health in later life: The Medical Research Council (MRC) National Survey of Health and Development. International Journal of Epidemiology 45, 1125–1134.

Gabry, J., Cesnovar, R., 2021. cmdstanr: R Interface to 'CmdStan'. URL: https://mc-stan.org/cmdstanr,https://discourse.mc-stan.org.

McElreath, R., 2020. Statistical Rethinking: A Bayesian Course with Examples in R and Stan. Texts in Statistical Science. 2nd ed., CRC Press, Boca Raton.

R Core Team, 2022. R: A Language and Environment for Statistical Computing. R Foundation for Statistical Computing. Vienna, Austria. URL: https://www.R-project.org/.

Stan Development Team, 2022. Stan modeling language: User's guide and reference manual, version 2.30 URL: https://mc-stan.org/docs/2_30/stan-users-guide-2_30.pdf.

Tuddenham, R.D., Snyder, M.M., 1954. Physical growth of California boys and girls from birth to eighteen years, in: Jones, H.E., Landreth, C., MacFarlane, J.W. (Eds.), University of California Publications in Child Development. University of California Press, Berkeley. volume 1, pp. 183–364.
